# Unifying duplication episode clustering and gene-species mapping inference

**DOI:** 10.1186/s13015-024-00252-8

**Published:** 2024-02-14

**Authors:** Paweł Górecki, Natalia Rutecka, Agnieszka Mykowiecka, Jarosław Paszek

**Affiliations:** Faculty of Mathematics, Informatics, and Mechanics, University of Warsaw, Banacha 2, Warsaw, 02-097 Poland

**Keywords:** Genomic duplication, Gene-species mapping, Duplication episode, Gene tree, Species tree

## Abstract

We present a novel problem, called MetaEC, which aims to infer gene-species assignments in a collection of partially leaf-labeled gene trees labels by minimizing the size of duplication episode clustering (EC). This problem is particularly relevant in metagenomics, where incomplete data often poses a challenge in the accurate reconstruction of gene histories. To solve MetaEC, we propose a polynomial time dynamic programming (DP) formulation that verifies the existence of a set of duplication episodes from a predefined set of episode candidates. In addition, we design a method to infer distributions of gene-species mappings. We then demonstrate how to use DP to design an algorithm that solves MetaEC. Although the algorithm is exponential in the worst case, we introduce a heuristic modification of the algorithm that provides a solution with the knowledge that it is exact. To evaluate our method, we perform two computational experiments on simulated and empirical data containing whole genome duplication events, showing that our algorithm is able to accurately infer the corresponding events.

## Introduction

In the field of computational biology, the use of gene families and the reconciliation model has become increasingly popular for studying the evolution of diverse organisms. These tools have facilitated the development of new algorithms and computational methods capable of handling large and complex datasets and exploring various types of evolutionary events. These events range from simple macroevolutionary processes such as gene duplications, gene losses, and horizontal gene transfers to more complex ones such as genomic duplications, speciations, and hybridizations. The reconciliation model has enabled researchers to reconstruct evolutionary histories by reconciling gene trees with species trees and identifying the evolutionary events that have led to the observed patterns of gene evolution. In the context of metagenomics, the reconciliation model has also been used to detect missing gene-species assignments using polynomial time algorithms. These developments have led to a better understanding of the evolutionary processes.

A classical reconciliation model [1, 2] defines a mapping from every node from a gene tree into a node in the species tree and determines if such a node is related to a speciation or can be classified as a single gene duplication [3]. In result, an embedding of the set of gene trees into a species tree can be interpreted as a joint evolutionary scenario [4]. The classical least common ancestor (LCA) mapping minimizes the number of single gene duplications and losses for one gene tree and the species tree [4].

The whole genome duplication (WGD) phenomenon incorporates additional copies of a complete genome into the original genetic material, thus creating an opportunity to introduce novel evolutionary traits [5, 6]. From a macro perspective, this phenomenon played a crucial role in the divergence and formation of species and shaped the evolution of almost all major lineages of life. In particular, many WGDs were uncovered in the evolutionary histories of plants, especially crops. WDGs potentially enabled the successful domestication of plants [7] and are important in the fight against famine [8]. Many traces and evidence of whole genome duplications can be found in the genomes of yeast and other fungal species [5, 9]. From the perspective of single cell evolution studies, WGDs are prevalent in cancer progression [10] and can lead us to the prognosis of advanced cancer stages [11] or the creation of strategies for targeted therapy [12].

Guigó et al. [13] proposed the first approach for detecting multiple gene duplication episodes from a collection of rooted gene trees. They designed a heuristic that aggregates single gene duplication events into a large gene duplication, given a collection of rooted gene trees and a rooted species tree. This approach was formalized and improved by Page and Cotton [14], who defined the problem of *episode clustering* ($\textsf{EC}$) as the task of identifying the minimal number of locations in the species tree where all duplications from the input gene trees can be placed. Fellows [15] applied this model in the context of the supertree problem. Polynomial-time solutions for two types of multiple gene duplication problems episode clustering and a more general variant of clustering called *minimum episodes* (ME) were proposed in [16, 17]. Luo et al. [18] proposed linear time and space algorithms, partially based on [19], for these problems. [20] introduced a unified approach by proposing a concept of interval models with a linear time and space solution to a broad class of clustering problems including EC and ME. Alternative approaches include generalization to unrooted gene trees; however, such approaches are often computationally complex [21, 22]. Other approaches include variants of clustering rules that depend on the maximal number of duplication episodes placed in one path [23, 24]. A comprehensive analysis of various models is available in [20]. Furthermore, [25] proposes an integer linear programming formulation that simplifies the process of testing these models. Relevant computational complexity results on the ME problem are presented in [26].

Metagenomic studies provide valuable information for analyzing entire communities of organisms and revealing a complete picture of their functional and adaptive capacities crucial for ecology [27] or human health [28]. Genetic material isolated in such studies can be used to detect whole genome duplication events.

One of the steps in metagenomic analysis is called binning. The aim of this procedure is to assign sequenced DNA fragments to the appropriate taxonomic groups. The assignment of certain genes to species can be ambiguous due to the limitations of annotation methods. A precise and comprehensive gene tree topology is essential for the accurate identification of potential duplication sites. The absence or misplacement of duplications in gene trees can, in turn, result in incorrect outcomes of methods aimed at determining whole genome duplication events.

To tackle the challenge of missing gene-species assignments in evolutionary studies, previous research has introduced methods based on the reconciliation score using gene duplication and loss events [29, 30]. In a related work, Mykowiecka et al. [31] extended this model by including horizontal gene transfer to better analyze bacterial evolution and proposed polynomial time algorithms for these models. These approaches utilize tree reconciliation according to the classical scheme, in which the gene tree includes symbols representing sequences with unclear species assignment in addition to the known gene labels. The objective is to assign the unknown gene labels to their corresponding species in a gene tree while minimizing the total reconciliation score, which is typically a weighted sum of evolutionary events such as gene duplication, gene loss, and horizontal gene transfer.

Here, we present a novel problem called MetaEC, which aims to infer gene-species assignments in a collection of gene trees with unknown labels by minimizing the size of episode clustering. This problem is particularly relevant in metagenomics, where incomplete data often poses a challenge in the accurate reconstruction of gene histories. To solve MetaEC, we propose a dynamic programming (DP) algorithm that verifies the existence of a set of duplication episodes from a predefined set of episode candidates. We then demonstrate how to use DP to design an algorithm that solves MetaEC. In addition, we design an algorithm to infer distributions of gene-species mappings from the set of all optimal solutions inferred by DP. Although the algorithm is exponential in the worst case, we introduce a heuristic modification of the algorithm that provides a solution with the knowledge that it is exact. To evaluate our method, we perform two computational experiments on simulated and empirical data containing WGD events, showing that our algorithm can accurately infer the corresponding events. It is important to note that a direct comparison of our novel algorithm with alternative methods is not possible, as there is currently no existing approach for the simultaneous inference of duplications and gene-species mappings.

## Basic definitions

### Gene trees, species trees, and the duplication cost

We begin by recalling some basic definitions from graph theory. All trees in this article are rooted and binary, therefore we refer to them as *trees*. For a tree $T=(V(T),E(T))$, by $\textsf{root} (T)$ we denote the root, and by *L*(*T*) we denote the set of all leaves. Every non-leaf node will be called *internal*. A *species tree* is a tree whose leaves are called *species*. A *gene tree* over a species tree *S* is a tree with leaves labeled by the species from *S*. The set of all species present in a species tree or a gene tree *T* is denoted by $\mathcal {L} (T)$. Note that for a species tree *S*, $L(S)=\mathcal {L} (S)$. Also, for a gene tree *G* over *S*, $\mathcal {L} (G) \subseteq L(S)$.

For nodes *a* and *b*, $a \preceq b$ means that *a* and *b* are on the same path from the root, with *b* being closer to the root than *a*. We write $a \prec b$ if $a \preceq b$ and $a \ne b$.

For a gene tree *G* over a species tree *S*, *the least common ancestor (lca) mapping* between *G* and *S* is a function $\textsf{M} _{G} :V(G) \rightarrow V(S)$ defined as follows. If *v* is a leaf in *G* then $\textsf{M} _G(v)$ is the label of *v*. When *v* is an internal node in *T* having two children *a* and *b*, then $\textsf{M} _G(v)$ is the least common ancestor of $\textsf{M} _G(a)$ and $\textsf{M} _G(b)$ in *S*. An internal node $g \in V(G)$ is called a *duplication* if $\textsf{M} _G(g) = \textsf{M} _G(a)$ for a child *a* of *g*. *The duplication cost*, denoted by $\textsf{D} (G,S)$, is the total number of duplications in *G*. Each non-duplication internal node of *G* we call a *speciation*. In the latter part of the article, a duplication *g* is called an *s*-duplication if $\textsf{M} _G(g)=s$. Similarly, we use the notation for an *s*-speciation and an *s*-leaf.

**Fig. 1:**
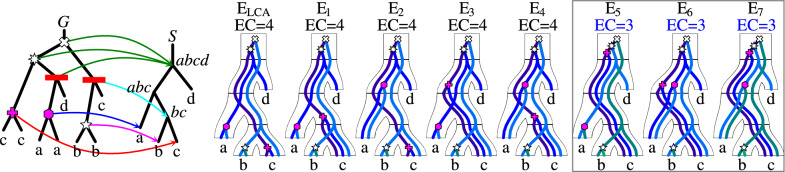
From the left side: a gene tree *G* and a species tree *S* with the lca-mapping M shown using arrows from the internal nodes of *G* to the nodes of *S*. There are 5 gene duplications, 2 speciation nodes in *G* (red bars), and 8 valid mappings depicted as embeddings of *G* into *S* [4], where the blue lines in these embeddings correspond to the edges of *G*. $E_{LCA}$ is induced by the lca-mapping. Here, EC (*G, S*) as depicted in the three rightmost scenarios with episode sets $\{a, b, abcd\}$ for $E_5$ and $\{a, abc, abcd\}$ for $E_6$ and $E_7$. The example trees are partially adapted from [24]

An example of tree reconciliation and the lca-mapping is depicted in the leftmost part of Fig. 1.

### Episode clustering problems

Below we present a model of duplication episodes proposed in [20]. In short, this model admits all evolutionary scenarios using duplication and loss events with a minimal number of gene duplications.

Formally, the model of gene duplication episodes allows for relocating a gene duplication from its lca-mapping node to one of its ancestors under some additional constraints required to preserve the biological soundness of the scenario. For a gene tree *G* over *S*, a mapping $\textsf{F} _G :V(G) \rightarrow V(S)$ is called *valid* if the following conditions are satisfied:

- $\textsf{F} _G(a) \preceq \textsf{F} _G(b)$ if $a \preceq b$ (time consistency),
- $\textsf{F} _G(a)=\textsf{M} _G(a)$ for any speciation node *a* (fixed speciations),
- $\textsf{F} _G(a) \succeq \textsf{M} _G(a)$ for any duplication node *a* (duplication can be raised),
- $\textsf{F} _G(a) \prec \textsf{M} _G(b)$ for any speciation node *b* such that $a \prec b$ (fixed number of gene duplications).

Note that the model of valid mappings described above is more comprehensive than the model presented in [16].

Figure 1 provides an example of valid mappings that uniquely define an evolutionary scenario that can be represented as a tree with an additional decoration of nodes. For more information on the formal modeling of evolutionary scenarios, refer to [4].

We denote by $\textsf{Dup} _T \subset V(T)$, the set of all duplication nodes in *T*. Let $G_1,G_2,\dots ,G_n$ be a collection of rooted gene trees over a species tree *S*. Assume that, for every $i \in \{1,2,\dots ,n\}$, $\textsf{F} _i$ is a valid mapping between $G_i$ and *S*. Every element $s \in \bigcup _i \textsf{F} _i[\textsf{Dup} _{G_i}]$^1^ denotes the location of multiple gene duplication events in *S*. We will refer to such locations as *duplication episodes* or simply *episodes*. Later on, we may also use the term episode to refer to the set of duplications that are mapped into it.

#### Problem 1

(Episode Clustering, EC) Given a collection of rooted gene trees $G_1,G_2,\dots ,G_n$ over a species tree *S*. Compute the minimal number of duplication episodes, denoted by $\textsf{EC} (G_1,G_2,\dots , G_n, S)$, in the set of all valid mappings $\textsf{F} _1,\textsf{F} _2,\dots ,\textsf{F} _n$ such that $\textsf{F} _i :V(G_i) \rightarrow V(S)$.

This problem can be solved in linear time and space [18].

### Gene-species mappings

We present the main problem for joint reconstruction of gene-species mappings and minimizing the set of episodes.

A *partial gene tree* is a rooted binary tree where each leaf is labeled by a species or has no label. Let *G* be a partial gene tree over *S*. By $\Lambda _G :L(G) \rightarrow L(S)$ we denote the partial *leaf labeling function* such that $\Lambda _G(g)$ is the label (species) of the leaf *g* in *G* if defined. Note that any gene tree is a partial gene tree with the leaf labeling being a total function. If a leaf in *G* has no label, we say that the label is *unknown* and write $\Lambda _G(g)=\bot$. We say that a gene tree $G^*$ over *S* *extends* a partial gene tree *G* over *S* if *G* and $G^*$ are isomorphic as graphs (i.e., $V(G)=V(G^*)$ and $E(G)=E(G^*)$), and, $\Lambda _{G^*}$ is a total function that extends $\Lambda _{G}$.

### Inferring labelings by minimizing episodes

Now, we present the problem of the simultaneous reconstruction of leaf labelings and duplication episodes from collections of partial gene trees.

#### Problem 2

(MetaEC) Given a collection of partial gene trees $G_1, G_2, \dots , G_k$ over a species tree *S*. Compute the minimum $\textsf{EC} (G^*_1, G^*_2, \dots , G^*_k,S)$, denoted $\textsf{EC} (G_1, G_2, \dots , G_k,S)$, in the set of all collections of gene trees $G^*_1, G^*_2, \dots , G^*_k$ such that $G^*_i$ extends $G_i$, for each *i*.

For example, if $(a,(\bot ,(\bot ,\bot )))$ is a single gene tree with three unknown labels and (*a*, (*b*, *c*)) is a species tree, then the problem is to replace all occurrences of $\bot$ by *a*, *b* or *c* such that the total number of duplication episodes is minimized. In this case, the optimal cost is 1, since at least one duplication is needed when the gene tree has four leaves and there are only three species.

## Methods

We begin by solving a simpler problem in which we assume that the set of duplication episodes is constrained to a given set of species tree nodes. Next, we show how to solve MetaEC for a single gene tree. Section MetaEC in the general case presents the general solution.

### Episode feasibility problem

We start with a related constrained problem. Given a partial gene tree, we are interested in the question of whether there is an extension of the partial gene tree such that the set of corresponding duplication episodes is contained in a given fixed set of episode candidates.

#### Problem 3

(Episode Feasibility) Given a partial gene tree *G* over a species tree *S* and $X \subseteq V(S)$. Does there exist a gene tree $G^*$ and a valid mapping $\textsf{F} _{G^*}$ such that $G^*$ extends *G* and $\textsf{F} _{G^*}(\textsf{Dup} _{G^*}) \subseteq X$?

If a partial gene tree *G* satisfies the above property, we call *G* *X*-*feasible* with respect to a species tree *S*. If the context is clear, we omit the reference to *S*.

The solution to Episode Feasibility is a dynamic programming (DP) formulation expressed using Łukasiewicz’s Three-Valued Logic Ł_3_ [32] with three constants $\textsf{True}$, $\textsf{False}$, and $\textsf{Unknown}$ (representing uncertainty) and ordered linearly: $\textsf{False}< \textsf{Unknown} < \textsf{True}$. The logic has binary operators $\vee$ (disjunction, $\max$), $\wedge$ (conjunction, $\min$), and two unary operators $\textsf{L}$ (certainty) and $\textsf{M}$ (possibility). See the interpretation in Table 1.

**Table 1:**
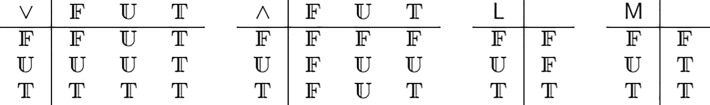
Boolean operations in Three-Valued-Log﻿ic

Here, $$\mathbb {F}=\textsf{False}$$, $$\mathbb {U}=\textsf{Unknown}$$, and $$\mathbb {T}=\textsf{True}$$

For a node *g* of a gene tree *G*, by *G*|*g* we denote the subtree of *G* rooted at *g*. For any non-leaf node *t* in a tree, by $t'$ and $t''$, we denote the children of *t*. To simplify the notation, we assume that the set $X \subseteq V(S)$ is fixed. Then, we have the following dynamic programming formulas that solve Episode Feasibility. Let $g \in V(G)$ and $s \in V(S)$.

$$\begin{aligned} \delta (g,s)= & {} \left\{ \begin{array}{ll} \delta ^*(g,s) &{} g \text { is internal and } s \in X, \quad \quad \quad \quad \qquad \qquad \qquad \qquad \quad \quad (1) \\ \delta ^*(g,s) \wedge \textsf{Unknown} &{} g \text { is internal and } s \notin X, \quad \quad \quad \quad \qquad \qquad \qquad \qquad \quad \quad (2)\quad \\ \textsf{False} &{} \text {otherwise,} \quad \quad \quad \quad \qquad \qquad \qquad \qquad \quad \quad \quad \qquad \quad \quad \quad \; (3) \end{array}\right. \\ \delta ^\downarrow (g,s)= & {} \left\{ \begin{array}{ll} \epsilon (g,s) &{} s \text { is a leaf,} \quad \quad \quad \quad \qquad \qquad \qquad \qquad \quad \quad \,\,\,\, (4) \\ \epsilon (g,s) \vee \textsf{M}\delta ^\downarrow (g,s') \vee \textsf{M}\delta ^\downarrow (g,s'') &{} s \text { internal, and } s \in X. \quad \quad \quad \quad \qquad \qquad \quad \, \;(5) \\ \epsilon (g,s) \vee \delta ^\downarrow (g,s') \vee \delta ^\downarrow (g,s'') &{} \text {otherwise,} \quad \quad \quad \quad \qquad \qquad \qquad \qquad \quad \quad \,\,\,\;(6) \end{array}\right. \\ \sigma (g,s)= & {} \left\{ \begin{array}{ll} \textsf{L}\big ( \delta ^\downarrow (g',s') \wedge \delta ^\downarrow (g'',s'') \vee \delta ^\downarrow (g',s'') \wedge \delta ^\downarrow (g'',s') \big ) &{} g \text { and } s \text { are internal,} \quad \quad \quad \,\, \;(7) \\ \textsf{True} &{} g \in L(G), \Lambda _G(g) \in \{s,\bot \}, \, \, \, \,\,(8) \\ \textsf{False} &{} \text {otherwise,} \quad \quad \quad \qquad \qquad \,\,\,\,\,\,\;(9) \end{array}\right. \end{aligned}$$

where

10 $$\begin{aligned} \epsilon (g,s)= & {} \sigma (g,s) \vee \delta (g,s), \end{aligned}$$

11 $$\begin{aligned} \delta ^*(g,s)= & {} \epsilon (g',s) \wedge \delta ^\downarrow (g'',s) \vee \epsilon (g'',s) \wedge \delta ^\downarrow (g',s). \end{aligned}$$

In the next Lemma, we express properties satisfied by the above formulas.

For a partial gene tree *G* over *S*, and nodes $g \in V(G)$ and $s \in V(S)$, we say that a valid mapping $\textsf{F} _T :V(G|g) \rightarrow V(S|s)$ is *feasible* for (*g*, *s*, *X*) if and only if *T* extends *G*|*g* and $\textsf{F} _{T}(\textsf{Dup} _{T}) \subseteq X$. Feasible mappings represent episode scenarios that correspond to partial solutions to the instance of Episode Feasibility that forces duplications from *G*|*g* to be present in episodes from $X \cap V(S|s)$.

We say a duplication *g* in a gene tree *T* is *upper* if the path from *g* to the root of *T* contains only duplications. The set of all upper duplications in a tree *T* is denoted by $\textsf{UDup} _T$. We say that a valid mapping $\textsf{F} _T :V(G|g) \rightarrow V(S|s)$ is *weakly feasible* for (*g*, *s*, *X*) if and only if there is no feasible mapping for (*g*, *s*, *X*), but there is *T* that extends *G*|*g*, *T* has at least one upper duplication *d* such that $F_T(d) \notin X$ and $\textsf{F} _{T}(\textsf{Dup} _T{\setminus }\textsf{UDup} _T) \subseteq X$. In contrast to feasible mappings, in weakly feasible mappings we constrain only non-upper duplications present in *G*|*g*. Here, the upper duplications are elements of episodes $s \notin X$. This situation is modeled by $\textsf{Unknown}$ value returned from $\delta ^\downarrow (g,s)$ and $\delta (g,s)$ calls, meaning that there is at least one duplication that needs to be assigned later (if possible) to an episode from $X \setminus V(S|s)$, which eventually occurs at levels of recursion shallower than the level of (*g*, *s*).

Informally, the meaning of DP formulas can be understood as follows. Below, let *T* be an extension of the subtree of *G* rooted at a node *g*. The value of $\delta (g, s)$ is $\textsf{True}$ if there exists *T* where *g* is an *s*-duplication and all duplications are assigned to the episodes from *X* (where $s \in X$ as well). Similarly, the value of $\sigma (g, s)$ is $\textsf{True}$ if there exists *T*, where *g* represents an *s*-speciation or an *s*-leaf node. Next, $\delta (g, s)$ is $\textsf{Unknown}$ if the condition for $\delta (g, s) = \textsf{True}$ is not met. However, there still exists *T*, where *g* represents an *s*-duplication, and all non-upper duplications from *T* are assigned to the episodes from *X*, while the upper duplications are assigned to episodes outside of *X*. It is important to note that in this case, *s* is not an element of *X*. Note that $\sigma (g, s)$ cannot be $\textsf{Unknown}$ since speciation nodes are fixed. Moving on, $\delta ^\downarrow (g, s)$ is $\textsf{True}$ if there exists *T* where all duplications are assigned to the episodes from *X*. Lastly, $\delta ^\downarrow (g, s)$ is $\textsf{Unknown}$ if the condition for $\delta ^\downarrow (g, s) = \textsf{True}$ is not met. However, there still exists *T*, where all non-upper duplications from *T* are assigned to the episodes from *X*, while the upper duplications are assigned to episodes outside of *X*.

While $\epsilon$ and $\delta ^*$ should be treated as “local” in the main formulas (i.e., they should not form separate arrays in implementation), their properties can be formulated as follows. Generally, if $\epsilon (g,s)$ is $\textsf{True}$, then there is a tree *T* where *g* is mapped into *s* and all duplications are assigned to the episodes from *X*. Since $\sigma (g,s)$ cannot be $\textsf{Unknown}$, $\epsilon (g, s)$ is $\textsf{Unknown}$ only if $\sigma (g,s)$ is $\textsf{False}$ and $\delta (g, s) = \textsf{Unknown}$. Again, here *g* is mapped to *s*. Now, $\delta ^*(g,s)$ is $\textsf{True}$ only if there is *T* where *g* is an *s*-duplication, and all duplication nodes below *g* are assigned to episodes from *X*. Importantly, at least one of the children of *g* must be mapped to *s*, which is captured by $\epsilon$. Furthermore, $\delta ^*(g,s)$ resembles $\delta (g,s)$, but the condition that duplications must be assigned to episodes from *X* only applies to the duplications (or upper-duplications) below *g* if $\delta ^*(g,s)$ is $\textsf{True}$ (or $\textsf{Unknown}$, respectively).

The following Lemma formalizes the conditions described above.

#### Lemma 1

Given a partial gene tree *G* over a species tree *S* and $X \subseteq V(S)$. Let $g \in V(G)$, $s \in V(S)$. Then,

1. $\delta (g,s)=\textsf{True}$ if and only if there is a gene tree *T* and a feasible mapping $\textsf{F} _T$ for (*g*, *s*, *X*) such that *g* is an *s*-duplication in *T*.
2. $\delta (g,s)=\textsf{Unknown}$ if and only if there is a gene tree *T* and a weakly feasible mapping $\textsf{F} _T$ for (*g*, *s*, *X*) such that *g* is an *s*-duplication in *T*.
3. $\sigma (g,s)=\textsf{True}$ if and only if there a gene tree *T* and a feasible mapping $\textsf{F} _T$ for (*g*, *s*, *X*) such that and *g* is an *s*-speciation or an *s*-leaf in *T*.
4. For any *g* and *s*, $\sigma (g,s) \ne \textsf{Unknown}$.
5. $\delta ^\downarrow (g,s)$ is $\textsf{True}$ if and only if there is a feasible mapping for (*g*, *s*, *X*).
6. $\delta ^\downarrow (g,s)$ is $\textsf{Unknown}$ if and only if there is a weakly feasible mapping for (*g*, *s*, *X*).

#### Proof

The proof is by induction on the structure of *G* and *S*. The base of induction is when $g \in L(G)$ and $s \in L(S)$, for which all properties are easy to verify.

*Inductive assumption:* For every *x*, *y* such that $g \succ x$, and $s \succeq y$ or $g \succeq x$ and $s \succ y$, P1-P6 are satisfied. *Inductive hypothesis:* For *g* and *s*, where at least one of *g* and *s* is not a leaf, P1-P6 are satisfied.

Additional notation: since functions are relations, we identify a function $f :A \rightarrow B$ with the sets of pairs $\{ (x,f(x) :x \in A \}$. If $\textsf{F} _{T'}:V(G|g') \rightarrow V(S|s)$ and $\textsf{F} _{T''}:V(G| g'')\rightarrow V(S|s)$ are valid mappings, then by $\textsf{F} _{T'}\oplus \textsf{F} _{T''}$ we denote the mapping $\textsf{F} _{T'}|_{V(T') {\setminus } \textsf{UDup} _{T'}} \cup \textsf{F} _{T'}|_{V(T'') {\setminus } \textsf{UDup} _{T''}} \cup \{(g,s)\} \cup (\textsf{UDup} _{T'} \cup \textsf{UDup} _{T''}) \times \{s\}$. Note that the resulting mapping is valid for a gene tree $(T',T'')$ that extends *G*|*g*.

We start with several properties.

(A1) if $\delta ^\downarrow (g',s)=\textsf{Unknown}$, then $s \notin X$. Assume that $s \in X$. Then, by P6, there is a weakly feasible mapping $\textsf{F} _{T'}$ for $(g',s,X)$. Now, let $\textsf{F} _{T'}':= \textsf{F} _{T'}|_{V(T') {\setminus } \textsf{UDup} _{T'}} \cup \textsf{UDup} _{T'} \times \{s\}$. It is not difficult to see that the mapping is feasible for $(g',s,X)$. A contradiction.

(A2) If $\epsilon (g',s)=\textsf{Unknown}$, then there is a weakly feasible $\textsf{F} _{T'}$ for $(g',s,X)$ and $g'$ is an *s*-duplication. Here, $\sigma (g',s)$ cannot be $\textsf{True}$, thus $\sigma (g',s)=\textsf{False}$, by P3 and P4. Therefore, $\delta (g',s)=\textsf{Unknown}$. The rest follows from P2.

We first prove properties P1-P4 for $\delta$ and $\sigma$. Then, we show that P5 and P6 hold for *g* and *s*.

(P1, $\Rightarrow$): If $\delta (g,s)=\textsf{True}$, then, from (1) *g* is internal, $s \in X$ and $\delta ^*(g,s) = \textsf{True}$. Then, w.l.o.g., for a child $g'$ of *g* $\epsilon (g',s) \wedge \delta ^\downarrow (g',s)=\textsf{True}$. Since, $\delta (g',s) \vee \sigma (g',s)$ is $\textsf{True}$, it follows from the inductive assumption for P1 and P3, that there is a feasible mapping $\textsf{F} _{T'}$ for $(g',s,X)$ such that $\textsf{M} _{T'}(g')=s$. For, the other child, we have $\delta ^\downarrow (g'',s)=\textsf{True}$. From P5, there is a feasible mapping $\textsf{F} _{T''}$ for $(g'',s,X)$. Now, let $T=(T',T'')$. Then, the mapping $\textsf{F} _{T'}\oplus \textsf{F} _{T''}$ is feasible for (*g*, *s*, *X*) and *g* is an *s*-duplication in *T*.

(P1, $\Leftarrow$): Assume there is a feasible $\textsf{F} _T$ for (*g*, *s*, *X*) such that $\textsf{M} _T(g)=s$ and *g* is a duplication in *T*. Since $g \in \textsf{UDup} _T$, we have that $s \in X$ and only (1) is satisfied. Thus, $\delta (g,s)=\delta ^*(g,s)$. W.l.o.g. we may assume that $M_{T}(g')=s$ (recall that *g* is a duplication). Then, $\textsf{F} _{T}|_{V(T|g')}$ is feasible for $(g',s,X)$. By the inductive assumption for P1 (if $g'$ is a duplication) or P3 (if $g'$ is a speciation or a leaf), we conclude that $\epsilon (g',s)=\textsf{True}$. For the second child, we have $M_{T}(g'')\preceq s$, thus $\textsf{F} _{T}|_{V(T|g'')}$ is feasible for $(g'',s,X)$ and by P5, $\delta ^\downarrow (g'',s)=\textsf{True}$. Finally, $\delta ^*(g,s)=\textsf{True} = \delta (g,s)$.

(P2, $\Rightarrow$): Let $\delta (g,s)=\textsf{Unknown}$. (Case P2.a) If $s \in X$ then, from (1) *g* is internal and $\delta ^*(g,s) = \textsf{Unknown}$. W.l.o.g., we may assume that $\epsilon (g',s) \wedge \delta ^\downarrow (g'',s)=\textsf{Unknown}$. Since $s \in X$, from (A1), we conclude that $\delta ^\downarrow (g'',s)=\textsf{True}$. Next, $\epsilon (g',s)=\textsf{Unknown}$ and by (A2) there is a weakly feasible mapping $\textsf{F} _{T'}$ for $(g',s,X)$ and $g'$ is an *s*-duplication. Since $s \in X$, similarly to the proof of *P*1, we can construct a feasible mapping for $(g',s,X)$ from weakly feasible $\textsf{F} _{T'}$. A contradiction. (Case P2.b) Assume that $s \notin X$. Then, from (2) *g* is internal and $\delta ^*(g,s) \wedge \textsf{Unknown} = \textsf{Unknown}$. We have that $\delta ^*(g,s) \in \{ \textsf{True}, \textsf{Unknown} \}$. (Case P2.b.1) Let $\delta ^*(g,s)=\textsf{True}$. W.l.o.g. assume that $\epsilon (g',s)=\delta ^\downarrow (g'',s) = \textsf{True}$. From P1 and $s \notin X$, $\delta (g',s)$ cannot be $\textsf{True}$, thus, $\sigma (g',s)=\textsf{True}$. From P3, there is a feasible $\textsf{F} _{T'}$ for $(g',s,X)$ and $\textsf{M} _{T'}(g')=s$. From P5, there is a feasible $\textsf{F} _{T''}$ for $(g'',s,X)$. Let $T=(T',T'')$, then $\textsf{F} _{T'}\oplus \textsf{F} _{T''}$, is weakly feasible for (*g*, *s*, *X*). Note that there is no feasible mapping for (*g*, *s*, *X*), which follows from the fact that $g'$ is an *s*-speciation, thus *g* is an *s*-duplication and $s \notin X$.

(Case P2.b.2) Let $\delta ^*(g,s)=\textsf{Unknown}$. W.l.o.g. assume that $\epsilon (g',s) \wedge \delta ^\downarrow (g'',s) = \textsf{Unknown}$. If $\epsilon (g',s)=\textsf{True}$ then $\delta ^\downarrow (g'',s)=\textsf{Unknown}$. Then, similarly to the previous case $g'$ is an *s*-speciation and there is a feasible $\textsf{F} _{T'}$ for $(g',s,X)$, while, from P6, there is a weakly feasible $\textsf{F} _{T''}$ for $(g'',s,X)$. Then, similarly to the previous case, let $T=(T',T'')$. Then, $\textsf{F} _{T'}\oplus \textsf{F} _{T''}$ is weakly feasible mapping for (*g*, *s*, *X*). It remains to analyse the case when $\epsilon (g',s)=\textsf{Unknown}$. By (A2) there is a weakly feasible $\textsf{F} _{T'}$ for $(g',s,X)$ and $g'$ is an *s*-duplication. Here, $\delta ^\downarrow (g'',s) \in \{\textsf{True},\textsf{Unknown} \}$, and depending on the value either, by P5 there is a feasible ($\textsf{True}$) or, by P6, weakly feasible ($\textsf{Unknown}$) $\textsf{F} _{T''}$ for $(g'',s,X)$. We conclude that $\textsf{F} _{T'}\oplus \textsf{F} _{T''}$ is weakly feasible mapping for (*g*, *s*, *X*). Since $g'$ is an *s*-duplication and $s \notin X$, there is no feasible mapping for (*g*, *s*, *X*) in this case.

(P2, $\Leftarrow$). Let $\textsf{F} _{T}$ be a weakly feasible mapping for (*g*, *s*, *X*) such that *g* is an *s*-duplication in *T*. Since *g* is an *s*-duplication, *g* is upper and $s\notin X$. Thus, $\delta (g,s)=\delta ^*(g,s) \wedge \textsf{Unknown}$ from (2). W.l.o.g. we may assume that $\textsf{M} _T(g')=s$ (note that *g* is an *s*-duplication). If $g'$ is a speciation or a leaf, then $\textsf{F} _T|g'$ is feasible for $(g',s,X)$ since no upper duplication is present in $T|g'$. From P3, $\sigma (g',s)=\epsilon (g',s)=\textsf{True}$. If $g'$ is a duplication then $g'$ is upper in $T|g'$, thus $\textsf{F} _T|g'$ is weakly feasible. From P2, $\delta (g',s)=\textsf{Unknown}$. In all cases, $\epsilon (g',s) \ge \textsf{Unknown}$. Similarly, for the second child of *g*, the mapping $\textsf{F} _T|g''$ is either weakly feasible for $(g'',s,X)$ if $g''$ is upper duplication outside *X*, or feasible otherwise. By P5 and P6, $\delta ^\downarrow (g'',s) \ge \textsf{Unknown}$. Finally, $\delta ^*(g,s) \ge \textsf{Unknown}$ and $\delta (g,s)=\delta ^*(g,s) \wedge \textsf{Unknown} = \textsf{Unknown}$.

(P3, $\Rightarrow$). Let $\sigma (g,s)=\textsf{True}$. Note that at least one of *g* and *s* is internal by the inductive assumption. Then, both nodes must be internal, from (7). W.l.o.g. we may assume that $\delta ^\downarrow (g',s') \wedge \delta ^\downarrow (g'',s'')=\textsf{True}$. Thus, from P5, we have two feasible mappings $\textsf{F} _{T'}$ for $(g',s',X)$ and $\textsf{F} _{T''}$ for $(g'',s'',X)$. Let $T=(T',T'')$, then *g* is an *s*-speciation and $\textsf{F} _{T'} \oplus \textsf{F} _{T''}$ is feasible for (*s*, *g*, *X*).

(P3, $\Leftarrow$). Assume that is a feasible mapping $\textsf{F} _T$ for (*g*, *s*, *X*) such that *g* is an *s*-speciation or an *s*-leaf in *T*. If *g* is a leaf, the statement is obvious. Assume that *g* is an *s*-speciation, then *s* is internal and $\sigma (g,s)$ follows from (7). W.l.o.g. we may assume that $\textsf{M} _{T}(g') \preceq s'$ and $\textsf{M} _{T}(g'') \preceq s''$. Thus, $\textsf{F} _{T}|_{V(T|g')}$ is feasible for $(g',s',X)$ since $\textsf{F} _T(V(T|g'))\subseteq V(S|s')$. From P5, $\delta ^\downarrow (g',s')=\textsf{True}$. Similarly, we obtain $\delta ^\downarrow (g'',s'')=\textsf{True}$. Finally, $\sigma (g,s) = \textsf{L}( \delta ^\downarrow (g',s') \wedge \delta ^\downarrow (g'',s'')) = \textsf{True}$.

(P4) It follows easily from the definition of $\sigma$ and the operator $\textsf{L}$.

(P5, $\Rightarrow$) Assume that $\delta ^\downarrow (g,s)=\textsf{True}$. (Case P5.1) If *s* is a leaf, then *g* is internal by the inductive assumption. Then, by (4), $\delta ^\downarrow (g,s)=\epsilon (g,s)=\delta (g,s) \vee \sigma (g,s) = \textsf{True}$. Note, that $\sigma (g,s)=\textsf{False}$, otherwise both *g* and *s* must be leaves. Thus, $\delta (g,s)=\textsf{True}$ and the feasible mapping for (*g*, *s*, *X*) exists by the already proven P1. (Case P5.2). If *s* is internal and $s \notin X$, then, by (6), $\delta (g,s) \vee \sigma (g,s) \vee \delta ^\downarrow (g,s') \vee \delta ^\downarrow (g,s'') = \textsf{True}$. If $\delta (g,s)=\textsf{True}$, there is a feasible mapping for (*g*, *s*, *X*) from already proven P1 for *g* and *s*. Similarly, we have the mapping from P3 if $\sigma (g,s)=\textsf{True}$. If $\delta ^\downarrow (g,s')=\textsf{True}$, then there is a feasible mapping $\textsf{F} _T$ for $(g,s',X)$ from the inductive assumption for P5. It should be clear that the mapping obtained from $\textsf{F} _T$ by enlarging the codomain from $V(S|s')$ to *V*(*S*|*s*) is feasible for (*g*, *s*, *X*). The remaining case when $\delta ^\downarrow (g,s'')=\textsf{True}$ is analogous. (Case P5.3). If *s* is internal and $s \in X$, then, by (5), $\delta (g,s) \vee \sigma (g,s) \vee \textsf{M}\delta ^\downarrow (g,s') \vee \textsf{M}\delta ^\downarrow (g,s'') = \textsf{True}$. The proof is the same as above if $\delta (g,s)=\textsf{True}$, $\sigma (g,s)=\textsf{True}$, $\delta ^\downarrow (g,s')=\textsf{True}$ or $\delta ^\downarrow (g,s'')=\textsf{True}$. For the remaining case, let $\delta ^\downarrow (g,s')=\textsf{Unknown}$, then there is a weakly feasible mapping $\textsf{F} _T$ for $(g,s',X)$ from the inductive assumption for P6. By placing all upper duplications from $\textsf{F} _T$ at episode *s* and enlarging the codomain from $V(S|s')$ to *V*(*S*|*s*), we construct a feasible mapping for (*g*, *s*, *X*). The remaining case when $\delta ^\downarrow (g,s'')=\textsf{Unknown}$ is analogous.

(P5, $\Leftarrow$) Assume there is a feasible mapping for (*g*, *s*, *X*). (Case P5.1) If *s* is a leaf, then *g* is internal by the inductive assumption. Thus, *g* is an upper *s*-duplication in *T*. Thus, $s \in X$ and by already proven P1, $\delta (g,s)=\textsf{True}$, $\epsilon (g,s)=\textsf{True}$ and $\delta ^\downarrow (g,s)=\textsf{True}$. (Case P5.2) Assume *s* is an internal node. If *g* is an *s*-duplication, then similarly to the above case, from P1, $s \in X$, and $\delta (g,s)=\textsf{True}$ and $\delta ^\downarrow (g,s)=\textsf{True}$ using (5). Analogously, we have the same conclusion, if *g* is an *s*-speciation (here, *s* does not have to be in *X*). For the remaining cases, there is $v \prec s$, such that *g* is either an *v*-duplication, or an *v*-speciation. W.l.o.g., we may assume that $v \preceq s' \prec s$. (Case P5.2.a) If *g* is an *v*-speciation, then $\textsf{F} _T(g)=v$ and there is no upper duplication in *T*. Since *T*|*v* is a subtree of $T|s'$, $\textsf{F} _T$ with a codomain $V(T|s')$ is feasible for $(g,s',X)$. From, the inductive assumption for P5, $\delta ^\downarrow (g,s')=\textsf{True}$ and also $\textsf{M}\delta ^\downarrow (g,s') = \textsf{True}$ (if $s \in X$). In all cases, $\delta ^\downarrow (g,s)=\textsf{True}$. (Case P5.2.b) If *g* is an *v*-duplication, then, from the feasibility of $\textsf{F} _T$, there is at least one node $w \in X$ on the path from *s* to *v* (there must be a duplication episode for *g*). Let *w* be the lowest (i.e., closest to *v*) node with the property. Now, we have two cases. If $w=s$, then $s \in X$, and there is no candidate in *X* for *g* below *s*, therefore, there is no the feasible mapping for $(g,s',X)$. However, there is a weakly feasible $\textsf{F} '_T$ for $(g,s',X)$ infered from $\textsf{F} _T$ by setting $\textsf{F} '_T(d):=s'$ all upper duplications *d* in *T* having $\textsf{F} _T(d)=s$, and $\textsf{F} '_T(u):=\textsf{F} _T(u)$ for the remaining nodes *u* from *T*. Clearly, $\textsf{F} '_T(V(T)) \subseteq V(S|s')$. Hence, from the inductive assumption P6, $\delta ^\downarrow (g',s)=\textsf{Unknown}$. Next, $\textsf{M}\delta ^\downarrow (g',s) = \textsf{True}$ and $\delta ^\downarrow (g,s)=\textsf{True}$ using (5). It remains to analyse the case when, $w \preceq s' \prec s$. Since, $\textsf{F} _T$ is $\preceq$-monotonic, $\textsf{F} _T(V(T)) \subseteq V(S|w) \subseteq V(S|s')$. Then, $\textsf{F} _T$ with the codomain shrinked to $V(S|s')$ is feasible for $(g,s',X)$. From the inductive assumption for P5, $\delta ^\downarrow (g,s')=\textsf{True}$. Here, it does not matter whether $s \in X$, in both cases we get $\delta ^\downarrow (g,s)=\textsf{True}$ from (5) or (6).

(P6, $\Rightarrow$) Assume, $\delta _X^\downarrow (g,s)$ is $\textsf{Unknown}$. Here, $\textsf{Unknown}$ is obtained only from (6), i.e., when $s \notin X$ and *s* is internal. We have $\delta (g,s) \vee \sigma (g,s) \vee \delta ^\downarrow (g,s') \vee \delta ^\downarrow (g,s'') = \textsf{Unknown}$. By P4, $\sigma (g,s)=\textsf{False}$. If $\delta (g,s)=\textsf{Unknown}$, then, we have a weakly feasible mapping from already proven P2. If $\delta ^\downarrow (g,s')=\textsf{Unknown}$, then, we have a weakly feasible mapping from the inductive assumption for P6. The same holds for $s''$.

(P6, $\Leftarrow$) Assume there is a weakly feasible mapping $\textsf{F} _T$ for (*g*, *s*, *X*). (Case P6.1) If *s* is a leaf, then *g* is internal by the inductive assumption. Thus, *g* is an upper *s*-duplication in *T*. Thus, $s \notin X$ and by already proven P2, $\delta (g,s)=\textsf{Unknown}$. Since, $\sigma (g,s)=\textsf{False}$, $\epsilon (g,s)=\textsf{Unknown}$ and $\delta ^\downarrow (g,s)=\textsf{Unknown}$ from (4). (Case P6.2) Assume *s* is internal. Note that *g* cannot be an *v*-speciation for any *v*, otherwise there is no upper duplication in *T* and $\textsf{F} _T$ cannot be weakly feasible. If *g* is an *s*-duplication in *T*, then similarly to the previous case, from P2, $s \notin X$, and $\delta (g,s)=\textsf{Unknown}$. Note that $\delta ^\downarrow (g,s')=\delta ^\downarrow (g,s'')=\textsf{False}$ (since $\textsf{M} _T(g)=s$). We conclude that $\delta ^\downarrow (g,s)=\textsf{Unknown}$ from (6). For the remaining case, there is $v \prec s$, such that *g* is an *v*-duplication. W.l.o.g., we may assume that $v \preceq s' \prec s$. From the weak feasibility of $\textsf{F} _T$, no node from the path between *s* to *v* (inclusively) is in *X*. Similarly, to P5.2, we construct weakly feasible $\textsf{F} '_T$ for $(g,s',X)$ from $\textsf{F} _T$ by setting $\textsf{F} '_T(d):=s'$ all upper duplications *d* in *T* having $\textsf{F} _T(d)=s$, and $\textsf{F} '_T(u):=\textsf{F} _T(u)$ for the remaining nodes *u* from *T*. By the inductive assumption for P6, $\delta ^\downarrow (g',s)=\textsf{Unknown}$. Next, if $v=s'$, then $\delta (g,s')=\textsf{Unknown}$ (since $x \notin X$) by P2, otherwise $\delta (g,s')=\textsf{False}$. Also, $\delta ^\downarrow (g,s'')=\textsf{False}$. This yields $\delta (g,s)=\textsf{Unknown}$ using (6). $\quad\quad\quad \square$

To solve Episode Feasibility, we have to apply $\delta ^\downarrow$ on the roots of the input trees.

#### Theorem 2

(Correctness) Given a partial gene tree *G* over a species tree *S* and $X \subseteq V(S)$. *G* is *X*-feasible if and only if $\delta _X^\downarrow (\textsf{root} (G),\textsf{root} (S))$ is $\textsf{True}$.

#### Proof

The proof follows immediately from P5 of Lemma 1: $\delta _X^\downarrow (\textsf{root} (G),\textsf{root} (S))$ is $\textsf{True}$ if and only if there is a feasible $\textsf{F} _T$ for $(\textsf{root} (G),\textsf{root} (S),X))$ such that *T* extends *G* and $\textsf{F} _T(\textsf{Dup} _T) \subseteq X$. $\quad\quad \square$

#### Theorem 3

(Complexity) Given a partial gene tree *G* over a species tree *S* and $X \subseteq V(S)$. The time and space complexity of solving Episode Feasibility by the dynamic programming algorithm is *O*(|*V*(*G*)||*V*(*S*)|).

#### Proof

We have three arrays $\delta _X$, $\delta ^\downarrow _X$ and $\sigma _X$ (note that $\epsilon$ and $\delta ^*$ can be directly inserted in their calls), each of size *O*(|*V*(*G*)||*V*(*S*)|) and every cell of an array can be computed in *O*(1) time. $\quad\quad\quad\quad\quad \square$

**Fig. 2:**
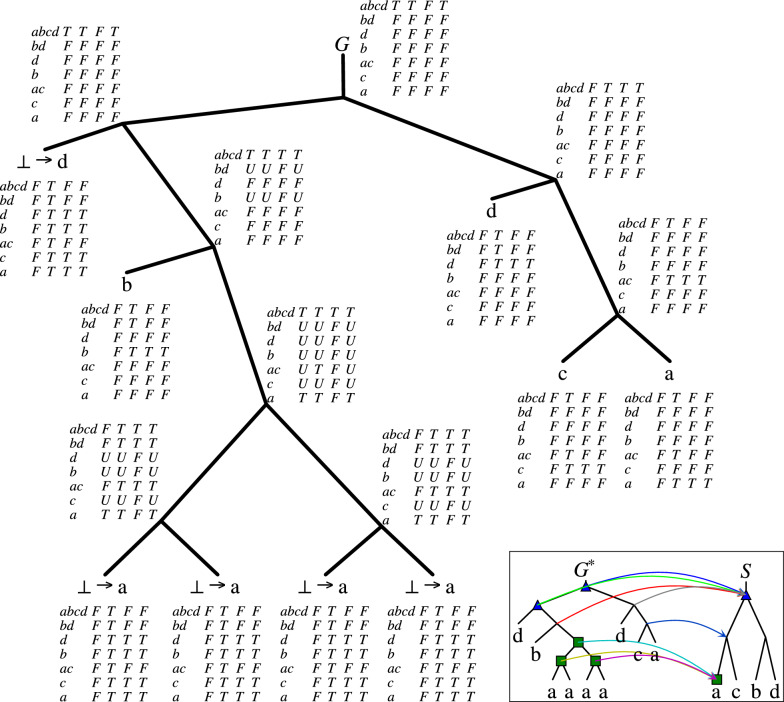
An example of the dynamic programming algorithm (from Sect. Episode Feasibility Problem) execution. The partial gene tree is $G=((\bot ,(b,((\bot ,\bot ),(\bot ,\bot )))),(d,(c,a)))$, which contains five unknown labels. The species tree, denoted as *S*, is represented as ((*a*, *c*), (*b*, *d*)). The marked nodes in *S* indicate episode candidates from *X*: the root of *S* (*abcd*) and the leaf node *a*. By applying dynamic programming, we obtain a feasible solution, depicted in the bottom-right corner. The resulting extension of the partial gene tree *G* is $G^*$, where the valid mapping between $G^*$ and *S* is the lowest common ancestor (LCA) mapping. In $G^*$, each duplication node is marked with a triangle or a square denoting their corresponding episode in *S*. Each node in *G* is decorated with an array that represents the values of DP formulas, where each row corresponds to a node in *S*, starting from *abcd*, *bd*, and so on as indicated in the first column. The next columns have the values of $\delta$, $\delta ^\downarrow$, $\sigma$, and $\epsilon$, respectively, for the gene tree node and the corresponding species tree node. For example, considering the root of *G* and the root of *S*, the top row of the array contains the following values: $\delta (\textsf{root} (G), \textsf{root} (S)) = \delta ^\downarrow (\textsf{root} (G), \textsf{root} (S)) = \epsilon (\textsf{root} (G), \textsf{root} (S)) = \textsf{True}$, while $\sigma (\textsf{root} (G), \textsf{root} (S)) = \textsf{False}$

An example of DP execution with a feasible solution is depicted in Fig. 2.

### Solving MetaEC for a single partial gene tree

Here we describe the main algorithm to solve MetaEC for instances with a single gene tree. First, we characterize an important property of episodes.

#### Lemma 4

(Fixed Episodes) Given a partial gene tree *G* over a species tree *S*. Assume that there are nodes *g* in *G* and *s* in *S* such that

- if *s* is the root of *S*, then at least one proper subtree of *G* contains species (leaf-labels) from both children of *s*.
- otherwise, let *p* be the parent of *s*, then *G*|*g* is a gene tree, *g* is a *p*-speciation and a child of *g* is an *s*-duplication.

Then, for any $G^*$ that extends *G*, *s* is an episode in every valid mapping between $G^*$ and *S*.

#### Proof

For the first case, all nodes above the root of the subtree are gene duplications mapped to the root of *S* in any $G^*$ that extends *G*. Therefore, the root of *S* is an episode in all valid mappings. In the second case, the duplication child cannot be raised, therefore, its mapping is fixed. $\square$

The nodes satisfying the above conditions we call *fixed episodes* (for *G* and *S*). For example, for trees from Fig. 1, there are two fixed episodes: the root of *S* and the leaf *b*, where the duplications with fixed mappings are depicted using white marks in the exemplary gene tree *G*. The set of all fixed episodes can be computed in linear time and space by bottom-up traversal of the partial gene tree *G* and by using LCA-queries in the species tree *S* as follows. For each node *g* from *V*(*G*), the algorithm computes a tuple (*u*, *s*, *d*), where

- $u \in \{\textsf{True},\textsf{False} \}$ is $\textsf{True}$ if and only if there is a leaf with unknown label reachable from *g*,
- $s \in V(S) \cup \{\textsf{None}\}$ is the least common ancestor of all non-$\bot$ labels reachable from *g* in *S* and $\textsf{None}$ if only $\bot$’s are visible from *g*,
- and $d \in \{\textsf{True},\textsf{False} \}$ is $\textsf{True}$ if and only if $u=\textsf{False}$ and *g* is a duplication node in a gene tree *G*|*g*.

Then, for each *g* and its tuple (*u*, *s*, *d*), and for each child of *g* with a tuple $(u',s',d')$:

- if $s=s'=\textsf{root} (S)$, then *s* (the root of *S*) is a fixed episode,
- if $u=d=\textsf{False}$, $d'=\textsf{True}$ and the parent of $s'$ is *s*, then $s'$ is a fixed episode.

We omit correctness and complexity proofs for brevity. Note that the number of fixed episodes is the lower bound of $\textsf{EC} (G,S)$.

Algorithm 1 takes as input a partial gene tree *G* over a species tree *S* and outputs $\textsf{EC} (G,S)$. It first computes the set of fixed episodes *F* (see Lemma 4). The algorithm then starts with an initial maximal episode number *b* equal to the number of nodes in *S*. In each iteration of a while loop, the algorithm checks if there is a set *C* of size $b-|F|-1$ from the vertices of *S* that are not in *F*, such that the partial gene tree *G* is $C \cup F$-feasible using the dynamic programming algorithm. This step requires $$\left( {\begin{array}{c}|V(S)|-|F|\\ b-|F|-1\end{array}}\right)$$ calls of DP in the worst case. If such a set *C* exists, the algorithm computes $\textsf{EC} (G^*,S)$ by the linear time algorithm from [20], where $G^*$ is the gene tree obtained by backtracking from the corresponding call of DP and updates *b* with the result. Note that *b* is not assigned the value of $|C \cup F|$, since the minimal set of episodes for $G^*$ and *S* is a subset of $C \cup F$, and it is often significantly smaller than $C \cup F$ in early steps of iteration. Updating *b* with $\textsf{EC} (G^*,S)$ guarantees the minimal number of episodes, where some elements of *C* may be unused. This is an important optimization step. If such a set *C* does not exist, the algorithm terminates and returns the current value of *b*.

**Algorithm 1:**
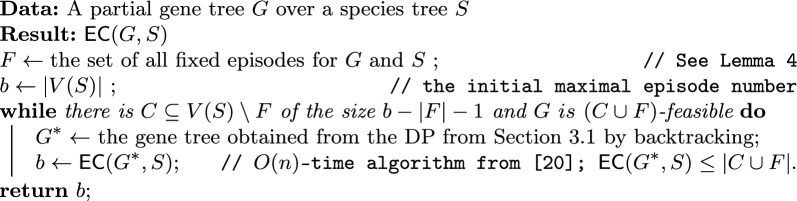
Solution to MetaEC with a single gene tree

The correctness of the algorithm follows from the fact that if there is no set *X* of size $b-1$ such that *G* is *X*-feasible, then there is no set of any size smaller than *b* that satisfies the property. Since *b* represents the number of episodes from some valid mapping, it is also minimal in such a case. Therefore, when the algorithm terminates, $b=\textsf{EC} (G,S)$, and the algorithm returns the correct value.

The algorithm’s worst-case time complexity is $$\sum _{k=f}^{n-f}{\left( {\begin{array}{c}n-f\\ k\end{array}}\right) nm} = O(nm2^n)$$, where *f* is the size of the set of fixed episodes ($f=|F|$), *n* denotes the number of vertices in *S*, and *m* denotes the number of vertices in *G*. Despite the exponential time complexity, in our experiments on both simulated and empirical data, we were able to compute exact solutions after only a few executions of the main loop.

#### Distributions of gene-species mappings

To evaluate the accuracy of gene-species mappings, we propose a method that enhances the DP algorithm by incorporating formulas for inferring the number of gene-species mappings present in all feasible reconstructed mappings. These counts can be collectively integrated to determine, for any leaf with unknown label, the precise frequency of its mappings to each species leaf within these feasible mappings. An alternative method for approximating these frequencies was suggested in [31] using uniform sampling. However, in this work, we introduce an exact algorithm for this purpose.

For a fixed species tree *S*, and a node *g* from a partial gene tree *G*, we call a mapping $f :L(G|g) \times L(S) \rightarrow \{0,1,2,\dots \}$ a *counter on* *L*(*G*|*g*) if for every leaf *l* with unknown label, the sum $\sum _{s} f(l,s)$, denoted $\#f$, does not depend on *l*. Counters will be used to count how many times a given gene $\bot$-leaf is assigned to a species leaf in all feasible mappings. In such a case, $\# f$ is the number of all such feasible mappings. The counter fixed to a gene tree $\bot$-leaf *l* and represented by the function $f(l,\cdot )$, is referred to as the *gene-species distribution* (of *l*). Subsequently, we often examine normalized distributions, wherein each value is divided by $\# f$ (then $\sum _s f(l, s)=1$). For convenience counters also include other leaves, but their counts will be set to 0. We have the following basic counters:

- $\emptyset _A$ is the *zero counter on* *A*, i.e., the counter with $\# \emptyset = 0$,
- for *s* in *L*(*S*), $\textsf{B} _{l,s}$ is a counter on $\{l\}$ such that $\textsf{B} _{l,s}(l,s)=1$, and $\textsf{B} _{l,s}(l,s')=0$ for all $s' \ne s$.

Let $\oplus$ and $\otimes$ be commutative operators, where $\otimes$ has higher precedence that $\oplus$, satisfying the following properties.

- If *f* and *g* are counters on disjoint sets *A* and *B*, respectively, then $f \otimes g$ is the counter on $A \cup B$, such that for every $l \in A$, $(f \otimes g)(l,s)=f(l,s) \cdot \#g$.
- If *f* and *g* are counters on *A*, then $f \oplus g$ is the counter on *A*, such that, for every $l \in A$, $(f \oplus g)(l,s)=f(l,s) + g(l,s)$.

The empty counter is used when a part of a gene tree has no leaves with unknown labels, while the counter $B_{l,s}$ represents the situation when leaf *l* is assigned to *s*. Additionally, we use a special counter $\textsf{E}$ that represents situations where DP formulas return $\mathbb {F}$. The counter is evaluated as follows: $\textsf{E} \oplus f = f$ and $\textsf{E} \otimes f = \textsf{E}$ for any counter *f* including $\textsf{E}$.

By using the notation introduced in the dynamic programming algorithm, we define counters for DP $\delta _{\#\mathbb {T}}(g,s)$, $\delta _{\#\mathbb {U}}(g,s)$, $\delta ^\downarrow _{\#\mathbb {T}}(g,s)$, $\delta ^\downarrow _{\#\mathbb {U}}(g,s)$, $\epsilon _{\#\mathbb {T}}(g,s)$, $\epsilon _{\#\mathbb {U}}(g,s)$ and $\sigma _{\#}(g,s)$ to count distributions of gene-species mappings. E.g., $\delta _{\#\mathbb {T}}(g,s)$ is a counter for the leaves from *L*(*G*|*g*) that counts mappings for these leaves for the cases when $\delta (g,s)$ is $\textsf{True}$, and so on.

- In (1), $\delta _{\#\mathbb {T}}(g,s) = e_{\mathbb {T},\mathbb {T}} \oplus r$ where, $e_{i,j}=\epsilon _{\#i}(g', s) \otimes \epsilon _{\#j}(g'', s)$ and if $\textsf{sib} (v)$ is the sibling of a node *v*, $r=\bigoplus _{i,j \in \{\mathbb {T},\mathbb {U}\}, v \in \{g',g''\}, w \in \{s',s''\}} \epsilon _{\#i}(v, s) \otimes \delta ^\downarrow _{\#j}(\textsf{sib} (v),w)$.
- In (1–2) $\delta _{\#\mathbb {U}}(g,s) = e_{\mathbb {T},\mathbb {T}} \oplus e_{\mathbb {U},\mathbb {T}} \oplus e_{\mathbb {T},\mathbb {U}} \oplus e_{\mathbb {U},\mathbb {U}} \oplus r$, where, *r* and $e_{i,j}$ are defined above.
- In (10), $\epsilon _{\#\mathbb {T}}(g,s) = \sigma _{\#}(g,s) \oplus \delta _{\#\mathbb {T}}(g,s)$, and, if $\delta (g,s)=\textsf{Unknown}$, then $\epsilon _{\#\mathbb {U}}(g,s) = \delta _{\#\mathbb {U}}(g,s)$.
- In (4), $$\delta ^\downarrow _{\#\mathbb {U}}(g,s)=\epsilon _{\#\mathbb {U}}(g,s)$$ and $$\delta ^\downarrow _{\#\mathbb {T}}(g,s)=\epsilon _{\#\mathbb {T}}(g,s)$$.
- In (5), $$\delta ^\downarrow _{\#\mathbb {T}}(g,s)= \epsilon _{\#\mathbb {T}}(g,s) \oplus \delta ^\downarrow _{\#\mathbb {T}}(g,s') \oplus \delta ^\downarrow _{\#\mathbb {U}}(g,s') \oplus \delta ^\downarrow _{\#\mathbb {T}}(g,s'') \oplus \delta ^\downarrow _{\#\mathbb {U}}(g,s'')$$ and $$\delta ^\downarrow _{\#\mathbb {U}}(g,s)=\epsilon _{\#\mathbb {U}}(g,s)$$.
- In (6), $$\delta ^\downarrow _{\#\mathbb {T}}(g,s)= \epsilon _{\#\mathbb {T}}(g,s) \oplus \delta ^\downarrow _{\#\mathbb {T}}(g,s') \oplus \delta ^\downarrow _{\#\mathbb {T}}(g,s'')$$ and $$\delta ^\downarrow _{\#\mathbb {U}}(g,s)= \epsilon _{\#\mathbb {U}}(g,s) \oplus \delta ^\downarrow _{\#\mathbb {U}}(g,s') \oplus \delta ^\downarrow _{\#\mathbb {U}}(g,s'')$$.
- In (7), $$\sigma _\#(g,s) = \delta ^\downarrow _{\#\mathbb {T}}(g',s') \otimes \delta ^\downarrow _{\#\mathbb {T}}(g'',s'') \oplus \delta ^\downarrow _{\#\mathbb {T}}(g',s'') \otimes \delta ^\downarrow _{\#\mathbb {T}}(g'',s')$$.
- In (8), $\sigma _\#(g,s) = \textsf{B} _{g,s}$ if $g = \bot$, otherwise $\sigma _\#(g,s) = \emptyset _{\{g\}}$.
- In all remaining uncovered cases the counters are equal to $\textsf{E}$.

The following lemma states the crucial property of DP counters.

##### Lemma 5

(Correctness of counters for DP) Given a partial gene tree *G* and a species tree *S* and $X\subseteq V(S)$. *G* is *X*-feasible if and only if the counter $\delta ^\downarrow _{\#\mathbb {T}}(\textsf{root} (G),\textsf{root} (S))$ is not $\textsf{E}$ and for every leaf *l* of *G* with unknown label, $\delta ^\downarrow _{\#\mathbb {T}}(\textsf{root} (G), \textsf{root} (S))(l,s)$ is the number of all gene trees $G^*$ extending *G* such that $\textsf{F} _{G^*}$ is a valid mapping satisfying $\textsf{F} _{G^*}(\textsf{Dup} _{G^*}) \subseteq X$ and $\textsf{F} _{G^*}(l)=s$.

The proof of the above lemma follows by induction, similar to the proof of correctness of DP. We omit technical details.

**Fig. 3:**
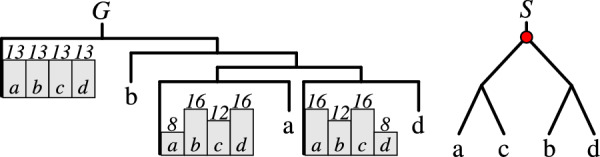
An example of gene-species distributions of for the leaves with unknown label from a partial gene tree with three such leaves and a species tree *S*. In this case, the optimal number of episodes is 1, and the episode is located at the root of *S*, marked accordingly. The total number of feasible mappings is 52. The histograms at the leaves of the gene tree depict how many times a specific leaf is mapped to the corresponding species leaf in these feasible mappings. In other words, they represent $\delta ^\downarrow _{\#\mathbb {T}}(\textsf{root} (G),\textsf{root} (S))(l,\cdot )$, where *l* is the gene tree leaf associated with each histogram

An example of gene-species distributions is depicted in Fig. 3. DP counters with verification algorithm are implemented in the software package metaEC.

#### Extensions

To identify the optimal solution within the main loop, enumerating all possible combinations of size $b-f-1$ from the set of episode candidates $V(S)\setminus F$ may be time-consuming for larger instances. To address this issue, we propose a heuristic approach that randomly samples combinations of size $b-f-1$ if $$\left( {\begin{array}{c}n-f\\ b-f-1\end{array}}\right)$$ is large (e.g., $>1000$) and adds a stopping condition based on the number of dynamic programming (DP) calls without improvement (e.g., after 100 calls). This approach not only speeds up the algorithm but also provides additional information on whether the returned value is exact or an upper bound obtained by switching to a heuristic mode. See Sect. 4 for more details.

Furthermore, based on our experiments, we have observed that the solution is often close to the set of fixed episodes. To leverage this observation, we propose a bottom-up algorithm that explores candidate sets starting from sizes 0, 1, 2, and so on until a feasible solution is found. In this case, the internal search has a time complexity of $$O(\left( {\begin{array}{c}n-f\\ i\end{array}}\right) )$$, starting from $i=0$. This algorithm can be combined with the heuristic variant described earlier to improve its effectiveness. However, the experimental evaluation did not show significant improvement compared to the top-down method in Algorithm 1.

### MetaEC in the general case

**Fig. 4:**
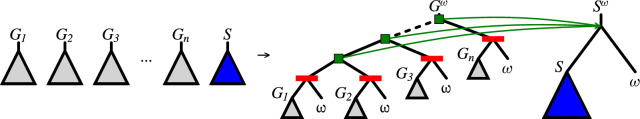
Converting a multiple gene tree instance to a single gene tree instance using an outgroup $\omega$. Red bars in $G^\omega$ denote speciation nodes mapped to the root of $S^\omega$. Green squares represent new duplications clustered at a new duplication episode in the root of $S^\omega$

Here we show that MetaEC in a general case can be solved using a single partial gene tree under an additional assumption. Given a collection of partial gene trees $G_1, G_2, \dots , G_k$ over a species tree *S*, let $\omega$ be a new species, called *outgroup*, not present in *S*. We first add the outgroup to every input tree. Let $S^\omega$ be species tree $(S,\omega )$, $G_1^\omega =(G_1,\omega )$ and $G^\omega _i=((G_i,\omega ),G^\omega _{i-1})$, for $i>1$. Then, by $\omega$-MetaEC we define the problem MetaEC with a single partial gene tree, where the extension of a partial labeling cannot introduce $\omega$, i.e., if $\Lambda _{G_1}(v)=\bot$ then $\Lambda _{G^*_1}(v) \ne \omega$. See Fig. 4 for illustration.

We have the following property.

#### Lemma 6

Given a collection of at least two partial gene trees $G_1, G_2, \dots , G_k$ over a species tree *S* such that $\omega \notin L(S)$. $X \subseteq V(S)$ is the set of episodes that yields the solution of MetaEC for $G_1, G_2, \dots , G_k$ and *S* if and only if $X \cup \{\textsf{root} (S^\omega )\}$ is the set of episodes that yields the solution to the instance $G^\omega _k$ and $S^\omega$ of $\omega$-MetaEC.

#### Proof

($\Rightarrow$) Assume that $G^*_i$ extends $G_i$, for each *i*. Then, $(G^*_i)^\omega$ extends $G_i^\omega$. Since the extension of $G_i$ introduces only nodes from *L*(*S*), we have the same property with $(G^*_i)^\omega$. Now, every parent of a leaf labeled $\omega$ in $(G^*_k)^\omega$ is speciation mapped to the root of $S^\omega$, since, for some *i*, its sibling is a root of $G^*_i$ and $\omega \notin \mathcal {L} (G^*_i)$. Thus, if $i>1$, the root of $(G^*_i)^\omega$ is a duplication mapped to the root of $S^\omega$. In summary, all duplications from gene trees $G^*_i$ in $(G^*_k)^\omega$ are mapped below the root of $S^\omega$, and they are separated by speciation nodes from the duplications mapped to the root of $S^\omega$ as indicated in Fig. 4. Now, we define a valid mapping $F^\omega _k = \textsf{F} _{(G^*_k)^\omega } :V((G^*_k)^\omega ) \rightarrow V(S^\omega )$. For each *i*, $\textsf{F} ^\omega _k$ on the set of nodes of $G_i$ equals the corresponding valid mapping between $V(G^*_i)$ and *V*(*S*) that yields the solution to MetaEC, while for the remaining nodes *v* we set $\textsf{F} _k^\omega (v) = \textsf{root} (S^\omega )$. It should be clear that $F^\omega _k$ is a valid mapping. Now, it is not difficult to see that the set of duplication episodes in $(G^*_k)^\omega$ is $X \cup \textsf{root} (S^\omega )$. If there is a solution to $\omega$-MetaEC with a lower number of episodes than $|X|+1$, say obtained by $X' \cup \{\textsf{root} (S^\omega )\}$ with $|X'|<|X|$, then the construction can be reversed to obtain valid mappings and the corresponding duplication episodes $X'$ for the initial instance of MetaEC. However, this is a contradiction with the assumption that |*X*| is the solution to the initial instance of MetaEC.

($\Leftarrow$) The proof of the second direction is analogous since the transformation between collections of partial gene trees and the partial gene tree $G^\omega _k$ is reversible. We omit easy details. $\quad\quad\quad\quad\quad \square$

Note that the algorithms provided in the previous sections can be easily modified to solve $\omega$-MetaEC, by replacing case (8) with:

$$\begin{aligned} \sigma (g,s)=\textsf{True} \text { if } g \in L(G) \text { and } (\Lambda _G(g)=s \text { or } (\Lambda _G(g)=\bot \text { and } s \ne \omega )). \end{aligned}$$

Then, DP will exclude extensions of $\bot$ by $\omega$.

## Experiments

In this Section we present two computational studies based on simulated and empirical data.

### Simulated dataset

The species trees having none, one, or two whole genome duplication events were taken directly from [25]. Then, we estimated gene trees via tree inference software from simulated sequences and modified them to represent the uncertainty commonly associated with metagenomic data. Finally, our algorithm was evaluated on six datasets consisting of estimated gene trees.

To begin, we describe how the species and gene trees were generated. Then, we explain the modifications made to the gene trees to represent the uncertainty of metagenomic data. Finally, we show how the results obtained with our algorithms allow us to infer genome-wide duplication events and gene and species distributions.

#### A species tree

First, we briefly summarize the simulation procedure from [25]. The simulated species trees were generated by SimPhy [33] with parameter settings used in a simulated study [34] that was based on an empirical dataset of 16 Fungi species [35]. The species tree *S* of 20 taxa was generated by SimPhy with the speciation rate parameter equal to $1.8 \times 10^{-9}$ and the tree height parameter set to $1.8 \times 10^9$.

**Fig. 5:**
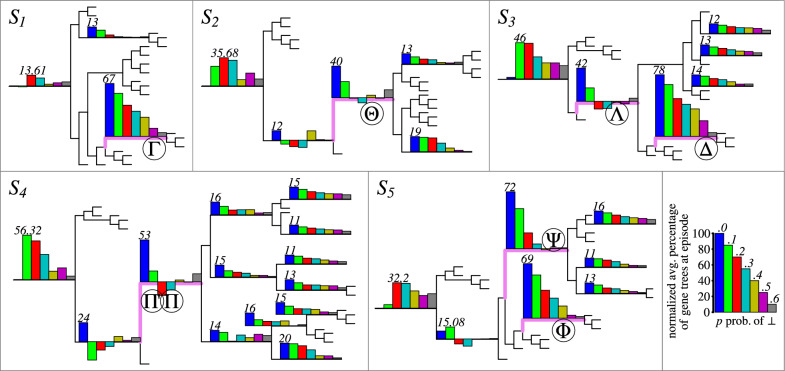
Summary of the inferred gene-species mappings and duplication episodes on the simulated datasets. Locations of the simulated whole-genome duplication (WGD) events are denoted by Greek letters. For clarity, all leaf labels have been removed from the visualization of species trees (see [25] for details). Each bar in the histograms shows the normalized average *p*-support of the corresponding species node. The key to histograms is present at the bottom-right corner. The number above a single bar represents the maximum height of a bar in its histogram. A histogram at node *v* is omitted as insignificant if the normalized average *p*-support is below 10 for all values of *p*

To simulate a whole genome duplication (WGD), a node *v* in the species tree *S* was chosen as the location of the event. Subsequently, a modified species tree, denoted as $S'$, was constructed by substituting a subtree *S*|*v* with a duplicated version of itself. This duplication involved creating a new root connected to the original root of *S*|*v* and the root of its copy. The WGD variants used in the simulations are illustrated in Fig. 5, where $S_1$ represents a single recent WGD $\Gamma$, $S_2$ represents a single ancient WGD $\Theta$, $S_3$ represents two WGDs $\Lambda$ and $\Delta$, with $\Delta$ occurring after $\Lambda$, $S_4$ represents two close WGDs $\Pi$ and $\Pi '$ at the same branch, and $S_5$ represents two recent independent WGDs $\Psi$ and $\Phi$. Let us refer to the original tree *S* with no WGD events as $S_0$. Note that these simulation methods do not incorporate fractionation, that is, the loss of a gene copy to eliminate the redundancy [36].

#### Gene trees

For every $S_i$, $i \in \{0, 1, \dots , 5\}$, one hundred *true gene trees* were generated using SimPhy. The duplication and loss rate parameter was set to $2^{-10}$ events per generation per lineage. To minimize the effect of incomplete lineage sorting, the population size parameter was set to 10. All other parameters were taken from [34].

Next, we describe our pipeline to infer *estimated trees* from true trees. For every true tree *G*, we first simulated a multiple sequence alignment (MSA).

For the MSA simulation, we used INDELible [37] and parameters from [34], with one difference, we used a constant sequence length of 1000. Then, we inferred an unrooted maximum-likelihood tree from each MSA using FastTree [38] with the GTR model. Finally, we performed midpoint-plateau rooting of each unrooted gene tree using URec [39]. The rooting was inferred in a way that minimizes the duplication-loss cost between the gene tree and the species tree *S*.

In summary, we obtained six datasets of estimated rooted gene trees denoted $\mathcal G_i=\{G_{i,1},G_{i_2},\dots ,G_{i,{100}}\}$, where each dataset comprises 100 gene trees generated using the same species tree *S* but with a different WGD scenario $S_i$. In our evaluation study, for each dataset $\mathcal G_i$, we generated a set of partial gene trees $\tilde{\mathcal G}^{k,p}_i$ by randomly removing each leaf label from every gene tree $G_{i,j}$ in $\mathcal G_i$ with probability *p*. We considered values of *p* from 0.0 to 0.6 in increments of 0.1 and generated 100 instances of $\tilde{\mathcal G}^{k,p}_i$ for each value of *p* and $k=1,2,\dots ,K$, with $K=100$. This resulted in a total of 4200 instances $(\tilde{\mathcal G}^{k,p}_i,S)$ of MetaEC, where *S* is the species tree used to generate $\mathcal G_i$, and $\tilde{\mathcal G}^{k,p}_i$ consists of 100 partial gene trees. Note that the instances for $p=0$, correspond to no removal of leaf labels, i.e., $\tilde{\mathcal G}^{k,0.0}_i=\mathcal G_i$, for each *i* and *k*.

**Fig. 6:**
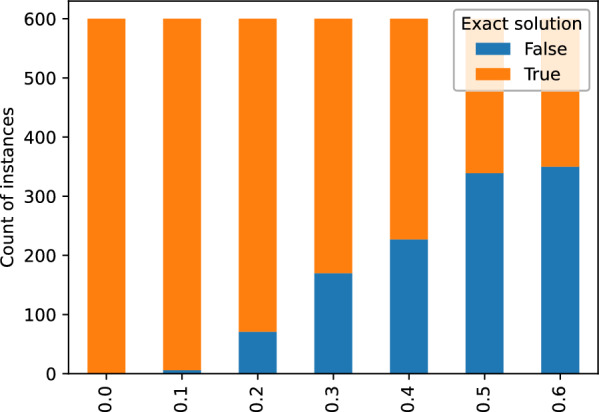
Summary of simulated dataset experiments for the estimated trees: histograms of exact and heuristic solutions returned by Algorithm 1

**Fig. 7:**
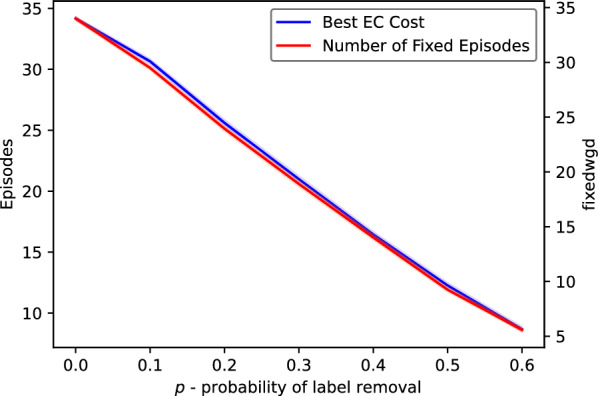
Summary of simulated dataset experiments for the estimated trees: $\textsf{EC}$ cost and the number of fixed episodes

**Fig. 8:**
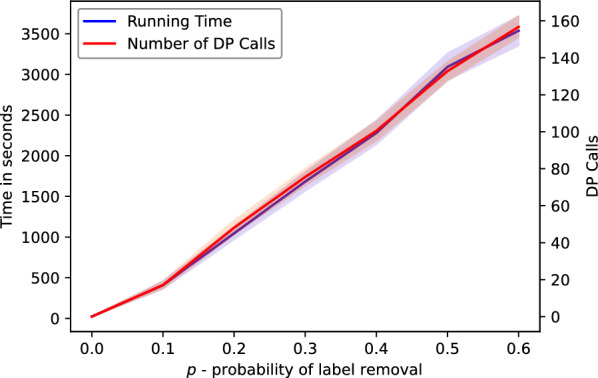
Summary of simulated dataset experiments for the estimated trees: the runtime in seconds and the number of executed DP calls

#### WGD detection

The results of our algorithm on the estimated partial gene trees are depicted in Fig. 5, where we summarize the episode sizes in the form of histograms. Also, additional data is provided in Figs. 6 and 7. The evaluation took about 24 h of a computing server with 80 cores. In general, we observed that the runtime and the number of DP calls grow linearly with the value of parameter *p* on average as indicated in the bottom diagram of Fig. 8. We used the heuristic variant of the algorithm, where random sampling was applied if the number of combinations exceeded 100 trees, and with the stopping criterion equal to 50. Out of 4200 instances, 3037 were completed with the exact solution (see Fig. 6). The resulting costs without exact guarantee, were more often obtained for larger values of *p*. Additionally, we observed that the lower bound given by the number of fixed episodes was a tight approximation of the inferred cost (see Fig. 8). As *p* increased, the number of fixed episodes decreased. Note that for $p=1$ (not included in our analysis), the solution to MetaEC is 1. In such a case the leaf labelings are constant functions and all internal nodes of each gene tree are duplications. Then there is just one episode, which can be placed at the root of the species tree.

Now, we briefly summarize the outcome of WGD detection. Initially in the preliminary version of the article [40], we quantified support by counting the number of single gene duplications aggregated within a particular episode. Here, to reflect the contributions of single gene families while mitigating the influence of large gene families with potentially numerous duplications, we count the number of gene trees that contain duplications clustered within the episode. Note that in contrast to [40] where the true trees were used to detect WGD events, here we perform the detection on estimated gene trees.

In the context of the scenario without WGDs ($S_0$), the episodes can be treated as background noise consisting of single duplication events. Consequently, when subtracting the contribution of background duplication episodes from the duplication clustering results on simulated datasets containing WGDs, we can emphasize the simulated WGD events as significant occurrences. Formally, our analysis is conducted using the following formulas.

Given a dataset $(\tilde{\mathcal G}^{k,p}_i,S)$ and a node *s* in *S*, the *p*-*support* of *s* (w.r.t. *k* and *i*), denoted $\mu _{p,s,i,k}$ is the number of gene trees obtained by applying the DP algorithm on trees from the dataset ${\mathcal G}^{k,p}_i$, that have at least one duplication in the episode *s*. Then, the *normalized average* *p*-*support of a species node* *s* *in scenario* $S_i$ is defined as

$$\begin{aligned} \frac{1}{K}\sum _k \mu _{p,s,i,k} - \mu _{p,s,0,k}. \end{aligned}$$

Recall that $K=100$ is the number of estimated gene tree datasets for fixed *p* and *i*. In other words, the normalized average support accounts for the difference between the average support values of $S_i$ and $S_0$. For example, given that every gene tree dataset consists of 100 estimated gene trees, the normalized average *p*-support of *s* in the scenario $S_i$ close to 100, denotes high support for the duplication episode at *s* (in $S_i$). Note also that the normalized *p*-support can be negative due to normalization.

The results for $S_1$ (as depicted in Fig. 5) reveal that WGD $\Gamma$ is uniquely well-supported when $p \le 0.4$, with support values exceeding 20. It is noteworthy that the outcomes obtained for $S_1$ outperform the analysis conducted in [40], which utilized true trees, in terms of accurately identifying the simulated WGD event. In summary, the WGD detection outcome depends on the location of a WGD and the value of *p*. In general, when *p* increases, the support of WGDs decreases. Recent WGDs like $\Gamma$, $\Delta$, and $\Phi$ are well supported up to $p=.3$, while more ancient $\Psi$ up to $p=.2$. For ancient WGDs $\Theta$, $\Lambda$, $\Pi$, $\Pi '$, the normalized average *p*-support is low and, for most cases, indistinguishable from non-WGD nodes. However, the root episode is generally well supported in scenarios having ancient WGDs, which suggest such an event close to the root location.

#### Gene-species distributions

We implemented the algorithm outlined in Sect. Distributions of gene-species mappings to derive counters with gene-species distributions for $\bot$-leaves across all gene tree datasets, i.e., for all six species trees $S_0$-$S_5$, all six different positive values of *p* (i.e., we inferred $36 \times 100$ counter collections for each gene tree set). The results frequently yielded remarkably high numbers of feasible mappings, often exceeding $10^{1000}$. As a remedy, we employed normalization by dividing the values of the gene-species distributions by $\#f$, where *f* was the relevant counter.

We first analyzed the domain of distributions with positive values. Let $d :L(S) \rightarrow [0,1]$ be a gene-species distribution. The span of *d* is the set of all species leaves with a positive value. Furthermore, if the span of this distribution is the set of leaves originating from a subtree rooted at *s* within the species tree *S*, we categorize this distribution as a *subtree-spanning distribution*. A summary of subtree-spanning and non-subtree-spanning distributions can be found in Table 2. It is noteworthy that spanning distributions occur with high frequency. Furthermore, they often encompass significant portions of the species tree, and the property gets stronger with the increase of *p*. This, however, is not the desired characteristic, as the most sought-after distributions are the one-point distributions where a single species leaf has the maximum value of 1.

**Fig. 9:**
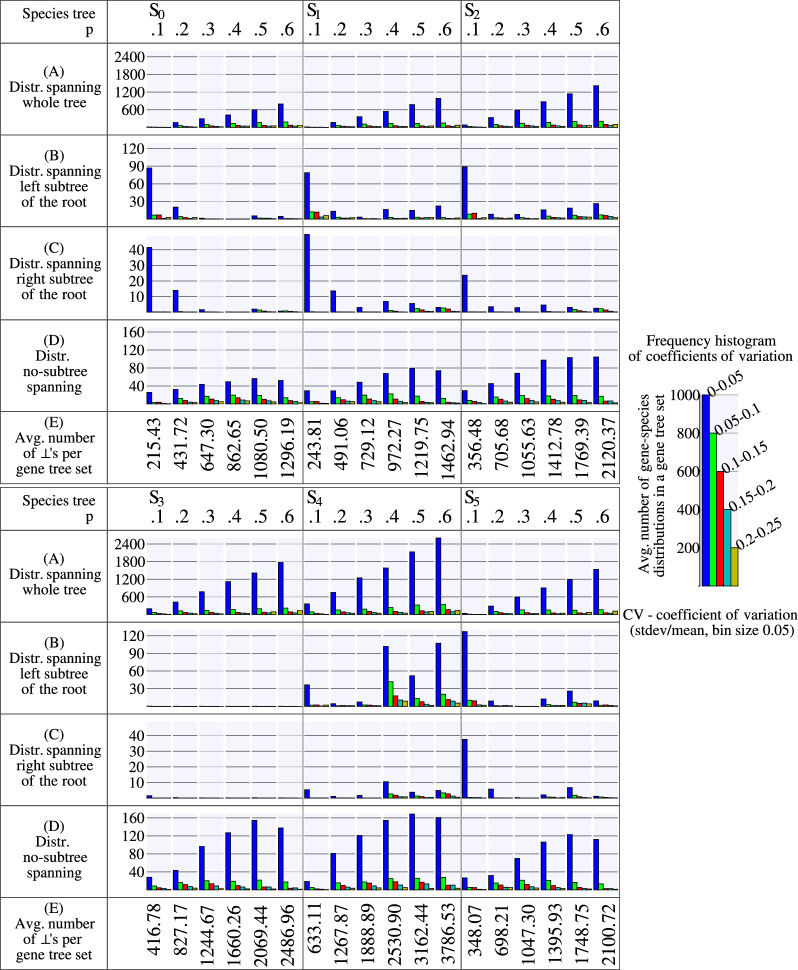
This figure summarizes coefficients of variations (CV) for gene-species distributions obtained from unlabeled leaves across 36 simulated datasets each with 100 gene trees (excluding datasets with $$p=0$$). Each bar corresponds to the average number of gene-species distributions for leaves with unknown label across the entire tree (**A**), the left subtree (**B**), and the right subtree (**C**), having the CV values falling within a specified range. All other distributions covering a subtree have all frequencies at most 1.5 and are therefore not included here. In **D**, a summary is presented for cases where the distributions do not span any subtree in the species tree. The panel (**E**) shows the average number of removed leaf-labels in the corresponding datasets. For example, the highest blue bar in $$S_4$$ with $$p=0.6$$ in **A** represents approximately 2400 leaves with $$\bot$$ (out of an average of 3786.53 in this gene tree set) whose mapping inferences give every leaf in $$S_4$$ with nearly identical frequency, as indicated by the corresponding CV values falling within the interval $$[0-0.05)$$. The key to histograms is on the right, where each bar represents the average count of gene-species distributions for $$\bot$$-leaves in a gene tree set with CV values falling within a specific interval. Intervals with CV values greater than 0.25 are excluded due to their low frequencies

Subsequently, we conducted an analysis to determine the extent to which these distributions deviate from uniformity. Detailed insights can be found in Fig. 9. In this figure, we present a computed coefficient of variation (CV) for each distribution, which is the standard deviation normalized by the mean. Lower CV values signify a closer resemblance to a uniform distribution. The majority of histograms display a tall blue bar indicating low CV values. This suggests that the distributions tended to exhibit a significant degree of near-uniformity in nearly all simulated datasets.

In summary, our study reveals that the species presented in the reconstructed gene leaf mappings extend across a substantial portion of the species tree. Additionally, the frequency distribution across all feasible mappings typically exhibits an almost uniform shape. As a result, identifying the true gene-species mapping signal within the leaf mapping proves challenging with the current approach. This challenge is, in part, attributed to the combinatorial explosion in the number of feasible mappings, consequently leading to flattened distributions of gene-species mappings based on leaf counts.

**Table 2:** Summary of spanning and no-spanning distributions of gene-species mappings for the simulated dataset

Dataset		Average	Subtree	No-subtree	Spanning			
Species tree	*p*	number	Spanning	Spanning	Root	Root left child	Root right child	Other nodes
		of $\bot$’s						
$S_0$	0.1	215.43	179.87	35.56	32.31	105.39	42.04	0.13
$S_0$	0.2	431.72	362.62	69.10	311.17	36.35	14.63	0.47
$S_0$	0.3	647.30	547.66	99.64	540.35	4.40	2.03	0.88
$S_0$	0.4	862.65	750.17	112.48	748.47	0.04	0.14	1.52
$S_0$	0.5	1080.50	973.41	107.09	951.94	14.58	4.80	2.09
$S_0$	0.6	1296.19	1206.18	90.01	1191.55	9.34	2.56	2.73
$S_1$	0.1	243.81	198.68	45.13	30.74	117.40	50.29	0.25
$S_1$	0.2	491.06	407.81	83.25	358.28	34.72	14.19	0.62
$S_1$	0.3	729.12	624.81	104.31	611.53	9.14	3.59	0.55
$S_1$	0.4	972.27	853.71	118.56	816.90	26.18	9.12	1.51
$S_1$	0.5	1219.75	1104.48	115.27	1059.34	32.34	10.78	2.02
$S_1$	0.6	1462.94	1363.53	99.41	1320.33	30.68	9.42	3.10
$S_2$	0.1	356.48	305.48	51.00	166.29	114.21	24.39	0.59
$S_2$	0.2	705.68	613.17	92.51	588.20	20.41	3.98	0.58
$S_2$	0.3	1055.63	934.53	121.10	914.00	16.33	3.31	0.89
$S_2$	0.4	1412.78	1268.31	144.47	1229.30	32.19	5.67	1.15
$S_2$	0.5	1769.39	1620.15	149.24	1569.99	41.02	6.78	2.36
$S_2$	0.6	2120.37	1976.13	144.24	1914.45	50.39	7.62	3.67
$S_3$	0.1	416.78	367.52	49.26	365.73	0.27	1.48	0.04
$S_3$	0.2	827.17	735.44	91.73	734.82	0.02	0.26	0.34
$S_3$	0.3	1244.67	1095.73	148.94	1094.94	0.01	0.14	0.64
$S_3$	0.4	1660.26	1490.09	170.17	1488.61	0.10	0.13	1.25
$S_3$	0.5	2069.44	1872.54	196.90	1869.82	0.14	0.21	2.37
$S_3$	0.6	2486.96	2316.49	170.47	2313.02	0.10	0.09	3.28
$S_4$	0.1	633.11	603.36	29.75	546.99	50.66	5.40	0.31
$S_4$	0.2	1267.87	1147.66	120.21	1135.46	10.60	1.29	0.31
$S_4$	0.3	1888.89	1717.59	171.30	1700.55	14.95	1.61	0.48
$S_4$	0.4	2530.90	2307.88	223.02	2100.00	188.21	17.67	2.00
$S_4$	0.5	3162.44	2923.63	238.81	2834.49	79.56	7.12	2.46
$S_4$	0.6	3786.53	3564.52	222.01	3386.89	160.26	13.52	3.85
$S_5$	0.1	348.07	301.20	46.87	74.72	186.24	38.68	1.56
$S_5$	0.2	698.21	615.30	82.91	582.68	26.10	6.01	0.51
$S_5$	0.3	1047.30	924.45	122.85	923.35	0.03	0.31	0.76
$S_5$	0.4	1395.93	1245.19	150.74	1218.45	21.38	3.87	1.49
$S_5$	0.5	1748.75	1593.88	154.87	1527.58	54.01	10.12	2.17
$S_5$	0.6	2100.72	1964.41	136.31	1941.82	16.87	2.77	2.95

### Empirical evaluation

To ensure that our algorithm was properly tested, we required a dataset that would capture the characteristics of the metagenomic data as closely as possible, while allowing us to assess the quality and accuracy of the results obtained. For this reason, we decided to prepare a dataset consisting of gene trees for species identified during metagenomic analysis. To simulate unknown gene-species assignments, we artificially removed some of the gene labels from the gene trees and retained information about their taxonomic origin for further analysis of the results. Another important issue was the presence of a previously described whole-genome duplication event that occurred in the evolutionary tree of selected species. Given the above requirements, we decided to use proteomes belonging to yeast species identified during metagenomic analysis of kefir [41]. Note that a direct comparison of our results with alternative methods is not possible due to the absence of any existing approach for the simultaneous inference of duplications and gene-species mappings.

**Fig. 10:**
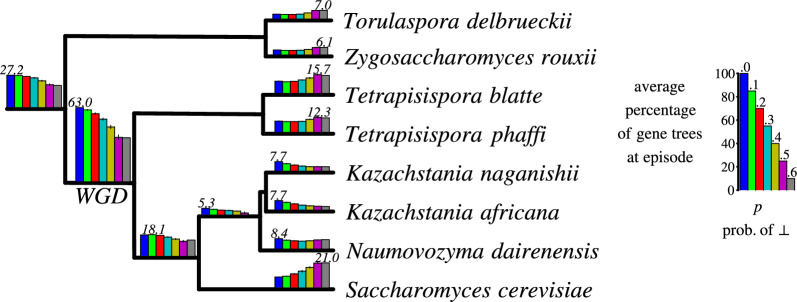
Summary of gene-species mappings and duplication episodes inference for the yeast dataset consisting of 3430 gene trees. Each bar with confidence levels represents the average percentage of the number of gene trees participating in the given duplication episodes at samples with value *p* from 0.0 to 0.6. WGD denotes the whole genome duplication event postulated in [42, 43]. For the description of symbols refer to Fig. 5

#### Data preparation

The eight selected species are: *Kazachstania Africana*, *Kazachstania naganishii*, *Naumovozyma dairenensis*, *Tetrapisispora blatte*, *Tetrapisispora phaffi*, *Torulaspora delbrueckii*, *Zygosaccharomyces rouxii* and *Saccharomyces cerevisiae*. A species tree containing the listed species, consistent with the NCBI taxonomy and many papers on yeast evolution, is shown in Fig. 10. It also shows the location of the whole-genome duplication event confirmed by previous studies [42, 43].

The proteomes used to infer gene trees were sourced from the UniProt database [44]. Protein families were created by dividing the proteins into groups using the *mcl* program [45] with parameters $I=2$ and $I=5$. However, since the differences between the obtained sets were minimal, we used the set obtained for $I=2$ in subsequent steps. The protein sequences in each group were aligned with the MUSCLE algorithm [46], and unrooted gene trees were inferred using the *phyml* program [47] with the default parameter setting. Rooting of the gene trees was performed by *URec* program [39] using the minimal duplication-loss cost as the rooting criterion.

We removed trees containing fewer than 3 leaves or 3 species, as well as trees with edges of length 0 from the final set of trees. This resulted in 3430 rooted gene trees. Similar to the first experiment, we created 10 datasets for each $p \in \{0.1, 0.2, \dots , 0.6\}$ by randomly removing each leaf label with the probability *p*. This resulted in 60 datasets plus the original dataset representing $p=0$.

#### Results

Figure 10 depicts histograms showing the results obtained for the described dataset. The evaluation was performed on the same computing server as before and took approximately one hour. For this evaluation, we set the sampling threshold and stopping criterion to 50, which yielded exact solutions for all cases. The number of fixed episodes was consistent across datasets with $p<0.6$, at 12 (note that the number of leaves in a tree was 15). For $p=0.6$, the number of fixed episodes fell within the range of 9 to 12. The number of DP calls ranged from 2 to 3 for $p<0.6$ and between 2 and 11 for $p=0.6$.

The results obtained by the algorithm for the yeast dataset are consistent with our knowledge of the whole-genome duplication localization. For the dataset with all leaf labels present and for $p=0.1$, we have the highest support for the WGD event. The number of supporting single duplications decreases gradually for successive $p's$. For values of $p \ge 0.5$, the correct WGD localization is still supported by a significantly large number of single duplications. It is worth noting that even for the $p=0.6$, the right location is supported by three times as many duplications as the second most supported location, which is in the root. Additionally, we observed an increase in duplications at the leaves of the species tree as *p* increased. Since most of the leaves are fixed episodes, the algorithm often assigned labels to create duplications at the leaves, resulting in larger sizes of episodes at leaves.

## Conclusions and future outlook

In this article, we presented a novel problem that integrates gene-species mapping inference and genomic duplication detection. We proposed efficient algorithms to solve the problem exactly in the majority of instances, along with a heuristic modification for cases where exact solutions are not feasible. To demonstrate the effectiveness and accuracy of our proposed algorithm, we conducted computational experiments on both simulated and empirical data. While there is presently no established method for the simultaneous inference of duplications and gene-species mappings, the results showed that our algorithm was able to accurately infer recent WGD events when the number of missing labels was relatively small for simulated data. Moreover, the algorithm performed even better on empirical data, demonstrating its robustness and applicability to real-world scenarios. Nevertheless, our findings regarding gene-species mapping inference underscore the challenging nature of the problem with the current approach. Inferring true gene leaf labels proves difficult due to the combinatorial explosion of potential solutions and the resulting nearly-uniform distributions of gene-species mappings, which extend across substantial portions of the species tree.

To maximize topological similarities between a gene tree and its species tree, speciation nodes should more frequently appear in the resulting extensions of input partial gene trees. We observe that the optimization model tends to reconstruct leaf labels in a way that prioritizes duplication events assigned to the nearest fixed episodes or the root, in the absence of such episodes. This is confirmed by the property that fixed episodes are tight approximations of the EC cost, leading to a reduction in the number of speciation events in the final gene tree extensions. As a consequence, the model’s effectiveness may be limited in some cases when the number of unknown labels in partial gene trees is significant.

Several avenues for future research exist. For instance, the simulation pipeline could be extended to model fractionation [36, 48], offering a more detailed understanding of the biological processes involved. Alternatively, tools for segmental duplications [49] might provide a more accurate representation of gene duplication processes. Also, we plan to extend the analyzed model to strengthen the importance of the topological similarities between gene and species trees. Alternatively, one may limit the distance between the lca-mapping of a gene duplication and its destination mapping in the final scenario similarly to [25]. Additionally, there are models of genomic duplications providing a higher level of detail than $\textsf{EC}$, such as minimum episodes (ME) [20] and RMP [23], which can be adapted in a similar way to infer gene-species mappings and minimize the number of duplication episodes simultaneously. These models can be further combined with more general models of valid mappings, which allow the introduction of more duplication events than the minimum obtained by the lca-mapping [4]. The combination of these models can provide a more comprehensive approach to inferring gene-species mappings and identifying the minimum number of duplication episodes.

## Data Availability

The software package metaEC, which is partially based on the embretnet repository, has been written in Python and is available with all datasets, including the commands to generate them and the output files, at https://bitbucket.org/pgor17/metaEC.

## Abbreviations

EC: Episode clustering
DP: Dynamic programming
WGD: Whole genome duplication
CV: Coefficient of variation
MSA: Multiple sequence alignment
ME: Minimum episodes

## Footnotes

1 Here, the notation *F*[*D*] denotes the image of *D* on the function *F*.

## References

[1] Goodman M, Czelusniak J, Moore GW, Romero-Herrera AE, Matsuda G (1979). Fitting the gene lineage into its species lineage, a parsimony strategy illustrated by cladograms constructed from globin sequences. Syst Zool.

[2] Page RDM (1994). Maps between trees and cladistic analysis of historical associations among genes, organisms, and areas. Syst Biol.

[3] Ma B, Li M, Zhang L (2000). From gene trees to species trees. SIAM J Comput.

[4] Górecki P, Tiuryn J (2006). DLS-trees: a model of evolutionary scenarios. Theoret Comput Sci.

[5] Kuzmin E, VanderSluis B, Ba ANN, Wang W, Koch EN, Usaj M, Khmelinskii A, Usaj MM, Leeuwen J, Kraus O, Tresenrider A, Pryszlak M, Hu M-C, Varriano B, Costanzo M, Knop M, Moses A, Myers CL, Andrews BJ, Boone C (2020). Exploring whole-genome duplicate gene retention with complex genetic interaction analysis. Science.

[6] Ohno S (1970). Evolution by gene duplication.

[7] Salman-Minkov A, Sabath N, Mayrose I (2016). Whole-genome duplication as a key factor in crop domestication. Nat Plants.

[8] Wu S, Lau KH, Cao Q, Hamilton JP, Sun H, Zhou C, Eserman L, Gemenet DC, Olukolu BA, Wang H, Crisovan E, Godden GT, Jiao C, Wang X, Kitavi M, Manrique-Carpintero N, Vaillancourt B, Wiegert-Rininger K, Yang X, Bao K, Schaff J, Kreuze J, Gruneberg W, Khan A, Ghislain M, Ma D, Jiang J, Mwanga ROM, Leebens-Mack J, Coin LJM, Yencho GC, Buell CR, Fei Z (2018). Genome sequences of two diploid wild relatives of cultivated sweetpotato reveal targets for genetic improvement. Nat Commun.

[9] Wolfe KH, Shields DC (1997). Molecular evidence for an ancient duplication of the entire yeast genome. Nature.

[10] López S, Lim EL, Horswell S, Haase K, Huebner A, Dietzen M, Mourikis TP, Watkins TBK, Rowan A, Dewhurst SM, Birkbak NJ, Wilson GA, Loo PV, Jamal-Hanjani M, Consortium T, Swanton C, McGranahan N (2020). Interplay between whole-genome doubling and the accumulation of deleterious alterations in cancer evolution. Nat Genet.

[11] Bielski CM, Zehir A, Penson AV, Donoghue MTA, Chatila W, Armenia J, Chang MT, Schram AM, Jonsson P, Bandlamudi C, Razavi P, Iyer G, Robson ME, Stadler ZK, Schultz N, Baselga J, Solit DB, Hyman DM, Berger MF, Taylor BS (2018). Genome doubling shapes the evolution and prognosis of advanced cancers. Nat Genet.

[12] Quinton RJ, DiDomizio A, Vittoria MA, Kotýnková K, Ticas CJ, Patel S, Koga Y, Vakhshoorzadeh J, Hermance N, Kuroda TS, Parulekar N, Taylor AM, Manning AL, Campbell JD, Ganem NJ (2021). Whole-genome doubling confers unique genetic vulnerabilities on tumour cells. Nature.

[13] Guigó R, Muchnik IB, Smith TF (1996). Reconstruction of ancient molecular phylogeny. Mol Phylogenet Evol.

[14] Page RDM, Cotton JA. Vertebrate phylogenomics: reconciled trees and gene duplications. Pacific Symposium on Biocomputing. 2002:536–47. 10.1142/9789812799623_0050 11928506

[15] Fellows M, Hallet M, Stege U. On the multiple gene duplication problem. In: 9th International Symposium on Algorithms and Computation (ISAAC’98), Lecture Notes in Computer Science 1533, Taejon, Korea, 1998:347–356.

[16] Bansal MS, Eulenstein O (2008). The multiple gene duplication problem revisited. Bioinformatics.

[17] Burleigh JG, Bansal MS, Wehe A, Eulenstein O. Locating multiple gene duplications through reconciled trees. In: Research in Computational Molecular Biology: 12th Annual International Conference, RECOMB 2008, Singapore, March 30-April 2, 2008. Proceedings 12, 2008:273–284. Springer

[18] Luo C-W, Chen M-C, Chen Y-C, Yang RWL, Liu H-F, Chao K-M (2011). Linear-time algorithms for the multiple gene duplication problems. IEEE/ACM Trans Comput Biol Bioinf.

[19] Mettanant V, Fakcharoenphol J. A linear-time algorithm for the multiple gene duplication problem. In: The 12th National Computer Science and Engineering Conference (NCSEC), 2008:198–203.

[20] Paszek J, Górecki P (2018). Efficient algorithms for genomic duplication models. IEEE/ACM Trans Comput Biol Bioinf.

[21] Paszek J, Górecki P (2016). Genomic duplication problems for unrooted gene trees. BMC Genomics.

[22] Paszek J, Górecki P. Inferring duplication episodes from unrooted gene trees. BMC Genomics. 2018;19(S5). 10.1186/s12864-018-4623-z PMC5998884 29745844

[23] Iersel LV, Janssen R, Jones M, Murakami Y, Zeh N. Polynomial-Time Algorithms for Phylogenetic Inference Problems involving duplication and reticulation. IEEE/ACM Trans Comput Biol Bioinf. 2019 10.1109/TCBB.2019.2934957 31425045

[24] Paszek J, Tiuryn J, Górecki P (2020). Minimizing genomic duplication episodes. Comput Biol Chem.

[25] Paszek J, Markin A, Górecki P, Eulenstein O (2021). Taming the duplication-loss-coalescence model with integer linear programming. J Comput Biol.

[26] Dondi R, Lafond M, Scornavacca C (2019). Reconciling multiple genes trees via segmental duplications and losses. Algorithms Mol Biol.

[27] Royo-Llonch M, Sánchez P, Ruiz-González C, Salazar G, Pedrós-Alió C, Sebastián M, Labadie K, Paoli L, Ibarbalz FM, Zinger L, Churcheward B, Coordinators TO, Chaffron S, Eveillard D, Karsenti E, Sunagawa S, Wincker P, Karp-Boss L, Bowler C, Acinas SG (2021). Compendium of 530 metagenome-assembled bacterial and archaeal genomes from the polar Arctic Ocean. Nat Microbiol.

[28] Wirbel J, Pyl PT, Kartal E, Zych K, Kashani A, Milanese A, Fleck JS, Voigt AY, Palleja A, Ponnudurai R, Sunagawa S, Coelho LP, Schrotz-King P, Vogtmann E, Habermann N, Niméus E, Thomas AM, Manghi P, Gandini S, Serrano D, Mizutani S, Shiroma H, Shiba S, Shibata T, Yachida S, Yamada T, Waldron L, Naccarati A, Segata N, Sinha R, Ulrich CM, Brenner H, Arumugam M, Bork P, Zeller G (2019). Meta-analysis of fecal metagenomes reveals global microbial signatures that are specific for colorectal cancer. Nat Med.

[29] Betkier A, Szczęsny P, Górecki P. Fast algorithms for inferring gene-species associations. In: Bioinformatics Research and Applications: 11th International Symposium, ISBRA 2015 Norfolk, USA, June 7–10, 2015 Proceedings 11, 2015:36–47. Springer.

[30] Zhang L, Cui Y. An efficient method for dna-based species assignment via gene tree and species tree reconciliation. In: Algorithms in Bioinformatics: 10th International Workshop, WABI 2010, Liverpool, UK, September 6–8, 2010. Proceedings 10, 2010:300–311. Springer.

[31] Mykowiecka A, Szczęsny P, Górecki P (2017). Inferring gene-species assignments in the presence of horizontal gene transfer. IEEE/ACM Trans Comput Biol Bioinf.

[32] Łukasiewicz J (1970). Selected Works.

[33] Mallo D, De Oliveira Martins L, Posada D (2016). Simphy: phylogenomic simulation of gene, locus, and species trees. Syst Biol.

[34] Molloy EK, Warnow T (2020). FastMulRFS: fast and accurate species tree estimation under generic gene duplication and loss models. Bioinformatics.

[35] Rasmussen MD, Kellis M (2012). Unified modeling of gene duplication, loss, and coalescence using a locus tree. Genome Res.

[36] Cheng F, Wu J, Cai X, Liang J, Freeling M, Wang X (2018). Gene retention, fractionation and subgenome differences in polyploid plants. Nat Plants.

[37] Fletcher W, Yang Z (2009). Indelible: a flexible simulator of biological sequence evolution. Mol Biol Evol.

[38] Price MN, Dehal PS, Arkin AP (2009). FastTree: computing large minimum evolution trees with profiles instead of a distance matrix. Mol Biol Evol.

[39] Górecki P, Tiuryn J (2007). Urec: a system for unrooted reconciliation. Bioinformatics.

[40] Górecki P, Rutecka N, Mykowiecka A, Paszek J. Simultaneous Reconstruction of Duplication Episodes and Gene-Species Mappings. In: Belazzougui D, Ouangraoua A, editors. 23rd International Workshop on Algorithms in Bioinformatics (WABI 2023), vol. 273. Leibniz International Proceedings in Informatics (LIPIcs). Dagstuhl, Germany: Schloss Dagstuhl—Leibniz-Zentrum für Informatik; 2023. p. 6–1618.

[41] Yilmaz B, Elibol E, Shangpliang HNJ, Ozogul F, Tamang JP (2022). Microbial communities in home-made and commercial kefir and their hypoglycemic properties. Fermentation.

[42] Feng B, Lin Y, Zhou L, Guo Y, Friedman R, Xia R, Hu F, Liu C, Tang J (2017). Reconstructing yeasts phylogenies and ancestors from whole genome data. Sci Rep.

[43] Marcet-Houben M, Gabaldón T (2015). Beyond the whole-genome duplication: phylogenetic evidence for an ancient interspecies hybridization in the baker’s yeast lineage. PLoS Biol.

[44] Consortium TU (2023). Uniprot: the universal protein knowledgebase in 2023. Nucleic Acids Res.

[45] Van Dongen S (2008). Graph clustering via a discrete uncoupling process. SIAM J Matrix Anal Appl.

[46] Edgar RC (2004). Muscle: a multiple sequence alignment method with reduced time and space complexity. BMC Bioinf.

[47] Guindon S, Dufayard J-F, Vincent L, Anisimova M, Hordijk W, Gascuel O (2010). New algorithms and methods to estimate maximum-likelihood phylogenies: assessing the performance of phyml 3.0. Syst Biol.

[48] Zhang Y, Zheng C, Sankoff D (2018). Pinning down ploidy in paleopolyploid plants. BMC Genomics.

[49] Davín AA, Tricou T, Tannier E, Vienne DM, Szöllősi GJ (2020). Zombi: a phylogenetic simulator of trees, genomes and sequences that accounts for dead linages. Bioinformatics.

